# Oncogenic Gα signaling requires AP-3-dependent recruitment to the endolysosomal compartment

**DOI:** 10.1073/pnas.2615774123

**Published:** 2026-07-22

**Authors:** Megha Shettigar, Salomé Moulière, Larissa Isenegger, Alexander L. DeVine, Cécile Gstalder, Vidyasagar Koduri, John G. Doench, Bruce Ksander, Mikel Garcia-Marcos, William G. Kaelin, Rizwan Haq

**Affiliations:** ^a^ Department of Medical Oncology, Dana-Farber Cancer Institute, Boston, MA 02115; ^b^ Department of Hematology, Brigham and Women’s Hospital, Boston, MA 02115; ^c^ Genetic Perturbation Platform, Broad Institute of Harvard and Massachusetts Institute of Technology, Cambridge, MA 02142; ^d^ Schepens Eye Research Institute of Massachusetts Eye and Ear, Boston, MA 02114; ^e^Department of Ophthalmology, Harvard Medical School, Boston, MA 02215; ^f^ Department of Biochemistry and Cell Biology, Chobanian and Avedisian School of Medicine, Boston University, Boston, MA 02118

**Keywords:** G-alpha, trafficking, uveal melanoma, lysosome, G protein

## Abstract

G protein–coupled receptors (GPCRs) constitute the largest superfamily of cell-surface receptors, yet the spatial constraints governing their signaling remain poorly defined. Here, we demonstrate that Gα, an essential downstream component of GPCR signaling, undergoes a spatial shift from the plasma membrane to the endolysosomal compartment upon its constitutive activation. Using a genome-wide CRISPR screen, we identify the adaptor protein AP-3 as the essential mediator of this trafficking event. Loss of AP-3-dependent recruitment impairs Gα-driven signaling and proliferation in uveal melanoma cells harboring oncogenic Gα mutations. We further identify a highly evolutionarily conserved AP-3 binding motif in all Gα proteins, present from yeast to humans. Disrupting this site causes Gα mislocalization and potently suppresses tumor growth and metastasis in vivo. Conversely, tethering Gα to the endolysosomal membrane is sufficient to rescue oncogenic signaling. Our findings reveal that endolysosomal recruitment is a fundamental, conserved requirement for Gα activity, uncovering a spatial vulnerability that may be exploited to target dysregulated G-protein signaling in human disease.

## Significance

G protein–coupled receptors and their associated G proteins are central regulators of human physiology and drivers of cancer, cardiovascular, neurological, and metabolic disorders. Although G-protein signaling was traditionally thought to originate at the plasma membrane, the spatial constraints governing active G-protein signaling have remained poorly understood. Here, we identify a regulator that recruits active Gα proteins to the endolysosomal compartment via an evolutionarily conserved motif shared across multiple Gα proteins. Disrupting this spatial recruitment potently suppresses tumor growth and metastasis in uveal melanoma, where Gα mutation is the dominant oncogenic event. These findings define a spatial vulnerability with broad therapeutic relevance for diseases driven by dysregulated G-protein signaling.

G protein–coupled receptors (GPCRs) are the largest superfamily of cell receptors in eukaryotes. They regulate numerous vital physiologic processes, including cell growth, immune responses, cardiovascular function, neurotransmission, and metabolic regulation. Reflecting its pathophysiologic importance, GPCR signaling is dysregulated in many cancers and other syndromes. For instance, more than 90% of uveal melanoma, 70% of appendiceal cancers, and a third of pituitary tumors have somatic mutations in components of GPCR signaling (1–8). Mutations in GPCR signaling proteins are also associated with congenital diseases, including osteodystrophy, blindness, and dystonias. Not surprisingly, over a third of approved drugs act on GPCRs, with many more being clinically investigated (9).

GPCRs transduce signals to heterotrimeric guanine nucleotide-binding regulatory proteins (G-proteins) consisting of α, β and γ subunits (10). GPCR agonism catalyzes the exchange of guanosine diphosphate (GDP) bound to the Gα subunit (inactive form) with guanosine triphosphate (GTP). This results in the activation of the Gα subunit and dissociation from the Gβγ heterodimer, enabling Gα and Gβγ to separately activate downstream effectors and thereby elicit cellular responses. The diverse cellular responses to GPCR activation depend, in part, on which of the four major classes of Gα proteins are activated. The Gα-mediated signal is terminated when its intrinsic GTPase activity hydrolyzes GTP into GDP (11, 12). To protect cells from overstimulation, GPCRs are regulated by multiple mechanisms, including phosphorylation by G protein–coupled receptor kinases (GRKs), interaction with β-arrestin, endocytosis-mediated downregulation, and inactivation of G-proteins by GTPase-activating proteins (GAPs) (13–22). Mutations at critical GTP-interacting residues within the Gα GTPase domain can impair its intrinsic GTPase activity, thereby locking the Gα subunit in a GTP-bound, constitutively active state. 90% of uveal melanoma patients have such mutually exclusive somatic mutations (Q209L/P) in *GNAQ* (Gα_q_) and *GNA11* (Gα_11_) within the GTPase domain, while similar mutations are found in Gα_s_ (R201) in pituitary and thyroid tumors and in Gα_i2_ (R179) in adrenocortical and ovarian tumors (1, 2, 23).

The subcellular localization of GPCR signaling may also influence physiologic effects. For example, while GPCRs and G-proteins classically signal at the plasma membrane and the cytoplasmic surface of the plasma membrane, respectively, numerous studies have noted their localization to subcellular compartments such as endosomes or the Golgi (20, 21, 24). Multiple studies also showed that some Gα_s_-mediated signaling processes occur specifically at early endosomes and the Golgi (25–32). Similarly, there have been some reports of Gα_q/11_ signaling at early endosomes, Golgi, and endoplasmic reticulum (33–40), and of Gα_i/o_ signaling at early and late endosomes, lysosomes, Golgi, and mitochondria (41–47). Based on these observations, it has been speculated that GPCR signaling in different subcellular compartments may elicit distinct signaling responses and physiologic effects (48). Nonetheless, the mechanisms that regulate the abundance and spatial bias of GPCR signaling components, as well as the relative contributions of intracellular membrane versus plasma membrane-localized GPCR signaling, are incompletely understood.

To comprehensively identify regulators of active Gα_q_ abundance and signaling, we performed a positive selection whole-genome CRISPR screen. We identified the AP-3 adaptor complex, which is involved in the trafficking of proteins to the lysosome, endosomes, and other lysosomal-related organelles (49–52), as an essential regulator of the abundance and subcellular localization of constitutively active Gα (hereafter referred to as Gα*). Gα* was found to be localized to the lysosome through a conserved AP-3-binding dileucine-based sorting signal, which is present in all members of the Gα family. Conversely, disruption of Gα* localization to the lysosome or the AP-3 binding site in Gα* led to diminished signaling and loss of ability to promote Gα*-dependent growth. Collectively, our studies identify a conserved molecular mechanism that regulates the spatial bias of Gα signaling and establish the functional importance of its localization to the endolysosomal compartment.

## Results

### Whole Genome CRISPR Screen Identifies AP-3σ as a Key Regulator of Constitutively Active Gα_q_ (Gα_q_*).

**Fig. 1.**
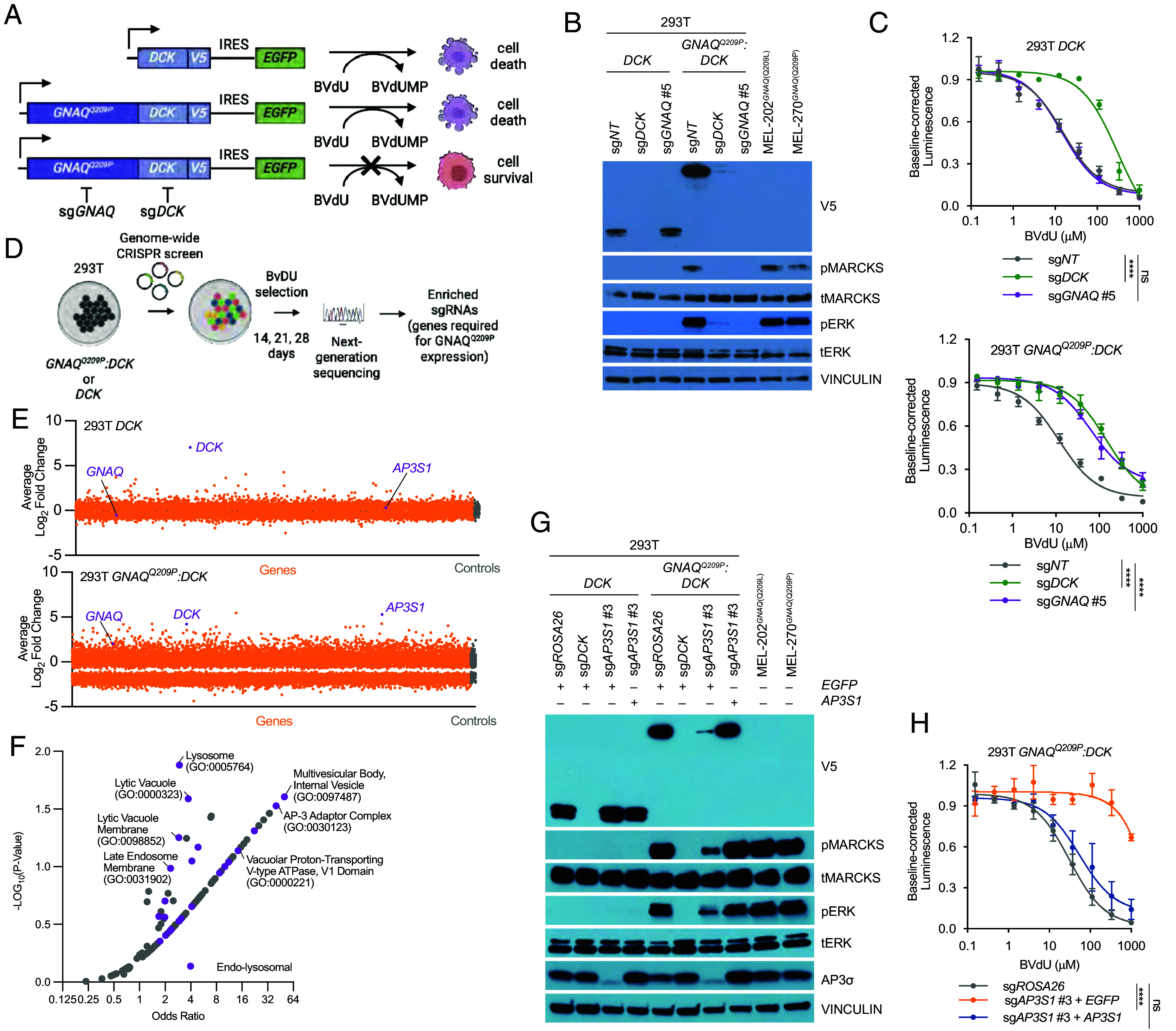
Whole genome CRISPR screen identifies AP-3σ as a key regulator of constitutively active Gα_q_ (Gα_q_*). (*A*) Schematic representation of the positive selection assay to identify regulators of Gα_q_*. *DCK*, variant of deoxycytidine kinase; V5, V5 epitope tag; IRES, internal ribosomal entry site; *EGFP*, enhanced green fluorescent protein; BVdU, bromovinyldeoxyuridine. (*B*) Immunoblot analysis of 293T *DCK* and 293T *GNAQ^Q209P^:DCK* cells transduced with sgRNAs targeting *DCK* and *GNAQ* or nontargeting controls (sg*NT*). (*C*) Baseline-corrected luminescence of 293T cells in (*B*) after 5 d of treatment with BVdU at the indicated concentrations. *n* = 3 biological replicates. (*D*) Schematic representation of the CRISPR-Cas9 positive selection screen performed in 293T *DCK* and 293T *GNAQ^Q209P^:DCK* cells transduced with the whole-genome Brunello sgRNA library. Following selection and 14 d of editing, cells were treated with BVdU for 28 d and harvested at 14, 21, and 28 d later to extract genomic DNA, which was sent for next-generation sequencing. (*E*) Average log_2_ fold change (*y*-axis) of sgRNAs after 28 d of BVdU treatment. Each dot represents the average of all sgRNAs targeting a specific gene. Gray dots represent nontargeting control sgRNAs. (*F*) Gene Ontology (GO) cellular component analysis of the top 100 genes enriched in the 293T *GNAQ^Q209P^:DCK* arm of the screen. (*G*) Immunoblot analysis of 293T *DCK* and 293T *GNAQ^Q209P^:DCK* cells transduced with indicated sgRNAs and/or reexpression of sgRNA-resistant *AP3S1*. (*H*) CellTiter-Glo analysis of 293T *GNAQ^Q209P^:DCK* cells transduced with indicated sgRNAs with or without *AP3S1* rescue after 5 d of treatment with BVdU at the indicated concentrations. *n* = 3 biological replicates. For (*C* and *H*), ordinary one-way ANOVA with multiple comparisons of AUC was used to determine statistical significance. ns, nonsignificant; *P < 0.05; ***P < 0.001; ****P < 0.0001. Error bars represent mean ± SEM.

We developed an assay to identify genes necessary for the abundance of constitutively active Gα_q_ (Gα_q_*). Screens performed to identify genes regulating the abundance of a given protein of interest often rely on readouts such as decreased protein abundance, decreased cell viability or decreased transcriptional activity. The results from such screens can be plagued by false positives that cause a general decrease in transcription or translation. In contrast, positive selection screens have a better signal-to-noise ratio and are therefore less vulnerable to false positives (53–56). Accordingly, we developed a positive-selection screening approach by fusing the constitutively active mutant of Gα_q_, *GNAQ^Q209P^*, to a modified form of deoxycytidine kinase (*DCK*) that converts the prodrug BVdU into a lethal toxin. Knockout of genes necessary for the expression of *GNAQ^Q209P^* or *DCK* thus renders the cells resistant to BVdU (positive selection), whereas cells expressing *GNAQ^Q209P^:DCK* or *DCK* are killed (Fig. 1*A*). We stably expressed either the *GNAQ^Q209P^:DCK* fusion or unfused *DCK* in 293T cells. FACS sorting was used to enrich for cells expressing high levels of the fusion protein based on concomitant EGFP expression (*SI Appendix*, Fig. S1*A*). Compared to *DCK*-expressing cells, *GNAQ^Q209P^:DCK*-expressing cells had elevated MAPK and PKC signaling (measured by pERK and pMARCKS, respectively), consistent with dysregulated Gα_q_ activity (57, 58) (Fig. 1*B*). Similarly, *GNAQ^Q209P^:DCK* expression activated YAP/TAZ signaling (59, 60) as determined by immunoblot and a YAP-dependent luciferase assay (*SI Appendix*, Fig. S1 *B* and *C*). As expected, cells expressing *GNAQ^Q209P^:DCK* or *DCK* were sensitive to BVdU, whereas the knockout of *DCK* conferred resistance to BVdU (Fig. 1*C*). Conversely, the knockout of *GNAQ* conferred BVdU resistance in *GNAQ^Q209P^:DCK* cells but not *DCK* cells. These differences in cell viability could not be attributed to a functional requirement of *DCK* or *GNAQ* in 293T cells since their deletion had no significant effect on cell growth in the absence of BVdU (*SI Appendix*, Fig. S1*D*).

We used these cells to identify genes that regulate the abundance of constitutively active Gα_q_. 293T *GNAQ^Q209P^:DCK* and *DCK* cells were infected with the Brunello whole-genome sgRNA library lentiviral supernatant encoding SpCas9 and sgRNAs targeting 19,114 genes, with approximately four guides per gene and 1,000 nontargeting guide RNAs (61) (Fig. 1*D*). After antibiotic selection and genome editing, a portion of the cells was harvested for genomic DNA isolation (T0), whereas the rest of the cells were treated with BVdU for 7 to 28 d. At weekly intervals, cells were harvested for the isolation of genomic DNA. Enrichment or depletion of sgRNAs was assessed by next-generation sequencing. We performed hypergeometric analysis to identify genes whose sgRNAs were enriched in the *GNAQ^Q209P^*: *DCK* and *DCK-*expressing cells upon BVdU treatment (“hits”) (Dataset S1). As expected, multiple sgRNAs targeting *DCK* were highly enriched in both arms of the screen, while a sgRNA targeting *GNAQ* was enriched only in the 293T *GNAQ^Q209P^:DCK* arm of the screen (Fig. 1*E* and *SI Appendix*, Fig. S2 *A*–*D*). While only one out of four sgRNAs targeting *GNAQ* was significantly enriched (*SI Appendix*, Fig. S2*D* and Dataset S1), we found that the remaining three *GNAQ* sgRNAs were simply ineffective in suppressing Gα_q_ abundance (*SI Appendix*, Fig. S2 *E*–*G*).

To identify common features of the top 100 enriched hits that exclusively scored in the 293T *GNAQ^Q209P^:DCK* arm of the screen and identify pathways that regulate Gα_q_* abundance, we performed enrichment analysis using the Enrichr tool (62–64). Interestingly, gene ontology analysis revealed that the top hits in the *GNAQ^Q209P^:DCK*-expressing cells were associated with the endolysosomal compartment or trafficking of proteins to the endolysosomal compartment (Fig. 1*F* and Dataset S2). In contrast, sgRNAs targeting genes related to retrograde trafficking were depleted (Dataset S1). Accordingly, we selected *AP3S1,* whose sgRNA was enriched exclusively in the *GNAQ^Q209P^:DCK* arm of the screen, for further validation and investigation (Fig. 1*E* and *SI Appendix*, Fig. S2 *H* and *I*). *AP3S1* encodes the σ3A subunit of the AP-3 heterotetrameric adaptor protein complex (65). The AP-3 adaptor complex is involved in the biogenesis of lysosome-related organelles and the sorting of cargo proteins to endosomes, lysosomes, and lysosome-related organelles (50, 66).

First, we tested by immunoblot analysis if *AP3S1* was necessary for *GNAQ^Q209P^:DCK* abundance and signaling to MAPK and PKC. Validating the screen results, knockout of *AP3S1* decreased Gα_q_^*^:DCK, pERK, and pMARCKS levels (Fig. 1*G*). *AP3S1* knockout cells were also, as expected, resistant to BVdU (Fig. 1*H*). These effects were on-target because they could be rescued by expressing a sgRNA-resistant *AP3S1* cDNA at endogenous expression levels (Fig. 1*G*) and that sensitivity to BVdU could be restored by reexpressing *AP3S1.*

The σ3 subunit of the AP-3 heterotetrameric adaptor protein complex has two isoforms, σ3A encoded by *AP3S1* and σ3B encoded by *AP3S2,* which share significant amino acid identity. The predicted molecular weights of σ3A and σ3B (together referred to as AP-3σ) are 21.73 and 22.01 kDa, respectively. Using an antibody that detects both proteins, we found that σ3A was more highly expressed (top prominent band) than σ3B (faint lower band) in 293T cells (Fig. 1*G*) (65). Furthermore, we observed that knocking out *AP3S1* with some, but not all, sgRNAs caused a reciprocal increase in the expression of the σ3B isoform (*SI Appendix*, Fig. S2*J*). To understand the specificity of the sgRNAs, we sequenced the genomic DNA surrounding the predicted editing sites. Sequencing data verified that the sgRNA that had the maximal effect on Gα_q_*:DCK abundance (sg*AP3S1* #3) targeted both *AP3S1* and *AP3S2*, likely because *AP3S2* shares 90% sequence identity with the *AP3S1* sequence at the editing site (*SI Appendix*, Fig. S3 *A* and *B*). These data suggest that the knockout of both *AP3S1* and *AP3S2* (sg*AP3S1/2)* is necessary for maximal effect on Gα_q_*:DCK abundance and signaling (*SI Appendix*, Fig. S4*A*). Consistent with this hypothesis, expression of the *AP3S2* cDNA was also sufficient to rescue Gα_q_*:DCK suppression in sg*AP3S1* #3-expressing cells (*SI Appendix*, Fig. S4 *B* and *C*).

### AP-3σ Is Necessary for Constitutively Active But Not Wild-Type Gα_q_ Abundance and Gα_q_*-Mediated Downstream Signaling.

**Fig. 2.**
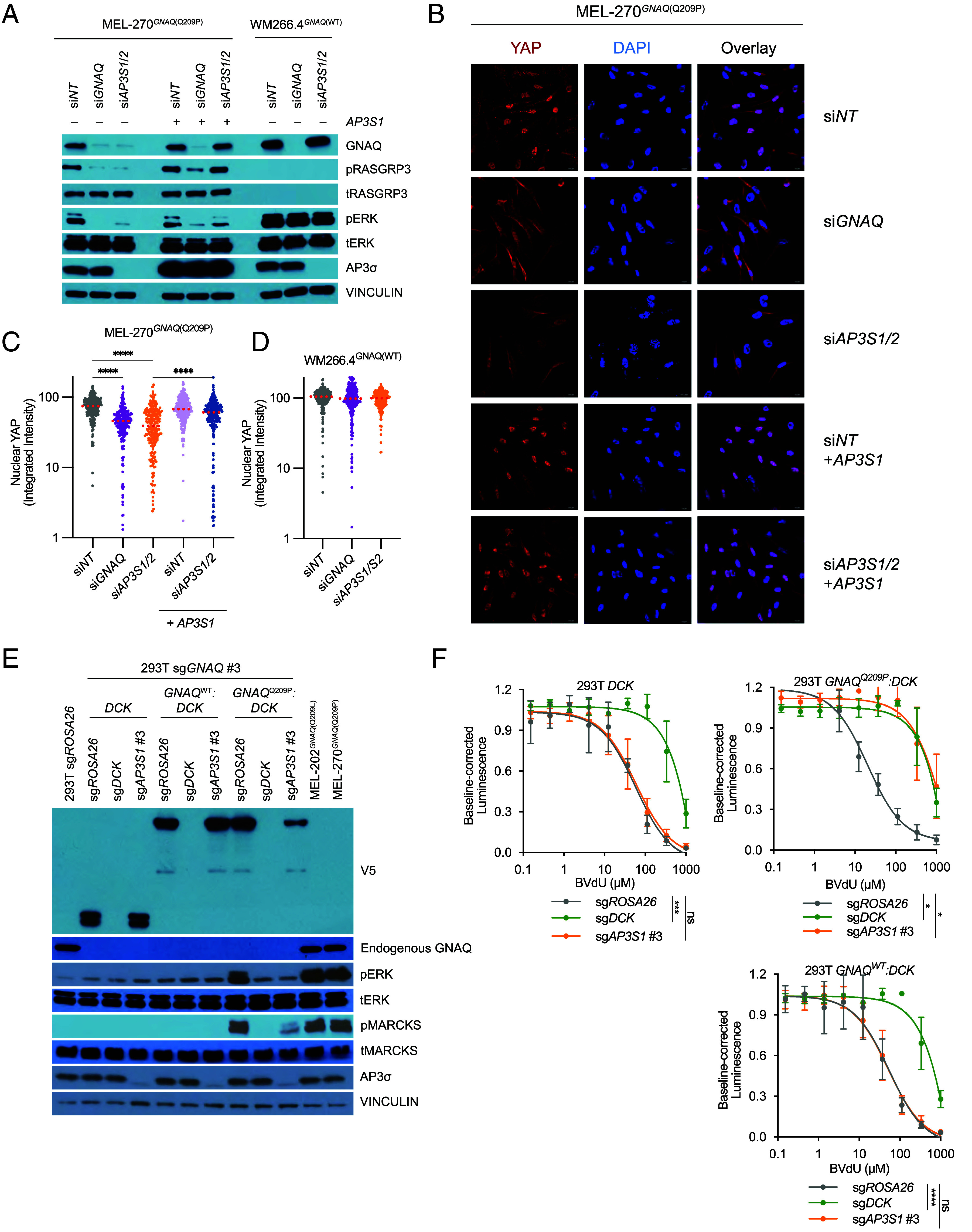
AP-3σ is necessary for constitutively active (Gα_q_*) but not wild-type Gα_q_ (Gα_q_^WT^) abundance and Gα_q_*-mediated downstream signaling. (*A*) Immunoblot analysis of MEL-270*^GNAQ^*^(Q209P)^ or WM266.4*^GNAQ^*^(WT)^ cells transfected with the indicated siRNAs and/or reexpression of siRNA-resistant *AP3S1*. (*B*) Immunofluorescence staining for endogenous YAP (red) in MEL-270*^GNAQ^*^(Q209P)^ cells from (*A*). (Scale bar, 25 μm.) (*C*) Intensity of nuclear YAP staining in MEL-270*^GNAQ^*^(Q209P)^ cells from (*B*). The dotted red line represents the mean intensity. *n =* 235 cells. (*D*) Intensity of nuclear YAP staining in WM266.4*^GNAQ^*^(WT)^ cells following transfection with siRNAs indicated. *n =* 235 cells. (*E*) Immunoblot of 293T sg*GNAQ*#3 cells expressing *DCK*, sg*GNAQ*#3 resistant *GNAQ^WT^:DCK,* and sg*GNAQ*#3 resistant *GNAQ^Q209P^:DCK* following editing by the indicated sgRNAs. (*F*) CellTiter-Glo analysis of cells from (*E*) after 5 d of treatment with BVdU at the indicated concentrations. *n* = 3 biological replicates. Ordinary one-way ANOVA with multiple comparisons of AUC was used to determine statistical significance for cell viability assays and ordinary one-way ANOVA with multiple comparisons for CellProfiler analysis. ns, nonsignificant; *P < 0.05; ***P < 0.001; ****P < 0.0001. Error bars represent mean ± SEM.

Next, we tested whether AP-3σ was necessary for the abundance of endogenous Gα_q_. We used the uveal melanoma cell line MEL-270, which harbors a *GNAQ^Q209P^* mutation, as well as a cutaneous melanoma cell line, WM266.4 (*GNAQ* wild-type). siRNA-mediated knockdown of *AP3S1* and *AP3S2 (*si*AP3S1/2*) in MEL-270*^GNAQ^*^(Q209P)^ cells significantly decreased Gα_q_* levels and downstream PKC and MAPK signaling (measured by pRASGRP3 and pERK, respectively), which was rescued by reexpressing a si*AP3S1-*resistant *AP3S1* cDNA. Knocking down *AP3S1* and *AP3S2* had no effect on Gα_q_^WT^ protein levels or MAPK signaling, while PKC signaling was not detectable in WM266.4*^GNAQ^*^(WT)^ cells (Fig. 2*A*).

We also evaluated the effect of knocking down *AP3S1* and *AP3S2* on YAP signaling in other cell lines that have different constitutively activating mutations in Gα_q_ or a different Gα protein, Gα_11_. *AP3S1/2* knockdown significantly decreased the intensity of nucleus-localized YAP, indicative of YAP activation in MEL-270*^GNAQ^*^(Q209P)^, MP41*^GNA11^*^(Q209L)^, and MEL-202*^GNAQ^*^(Q209L)^ cells, as determined by immunofluorescence and CellProfiler analysis. The intensity of nuclear YAP was rescued upon reexpression of *AP3S1* (Fig. 2 *A*–*C* and *SI Appendix*, Fig. S5 *A*–*F*). Knocking down *AP3S1* and *AP3S2* had no effect on YAP localization in WM266.4*^GNAQ^*^(WT)^ cells (Fig. 2 *A* and *D* and *SI Appendix*, Fig. S5*G*). These observations suggest that AP-3σ is selectively required for the abundance and signaling of constitutively active Gα_q_*.

To directly compare the effects of AP-3σ deficiency on Gα_q_^WT^ and Gα_q_* abundance and downstream MAPK and PKC signaling and to rule out the influence of endogenous Gα_q_^WT^ on the results of this experiment, we generated isogenic 293T cells expressing exogenous, sg*GNAQ*-resistant *GNAQ^Q209P^:DCK, GNAQ^WT^:DCK* or *DCK* and lacking endogenous *GNAQ* by virtue of CRISPR editing*. AP3S1* knockout decreased Gα_q_*:DCK but not DCK or Gα_q_^WT^:DCK levels (Fig. 2*E*). The decrease in Gα_q_*:DCK was accompanied by decreased pERK and pMARCKS levels. *AP3S1* knockout caused resistance to BVdU, but no changes in BVdU sensitivity in *DCK* and *GNAQ^WT^:DCK-*expressing cells (Fig. 2*F*). Collectively, our data show that AP-3σ is selectively required for the abundance and signaling of constitutively active Gα_q_ but not for the abundance of wild-type Gα_q_.

### Constitutively Active, But Not Unstimulated Wild-Type Gα_q_ Localizes to the Endolysosomal Compartment.

**Fig. 3.**
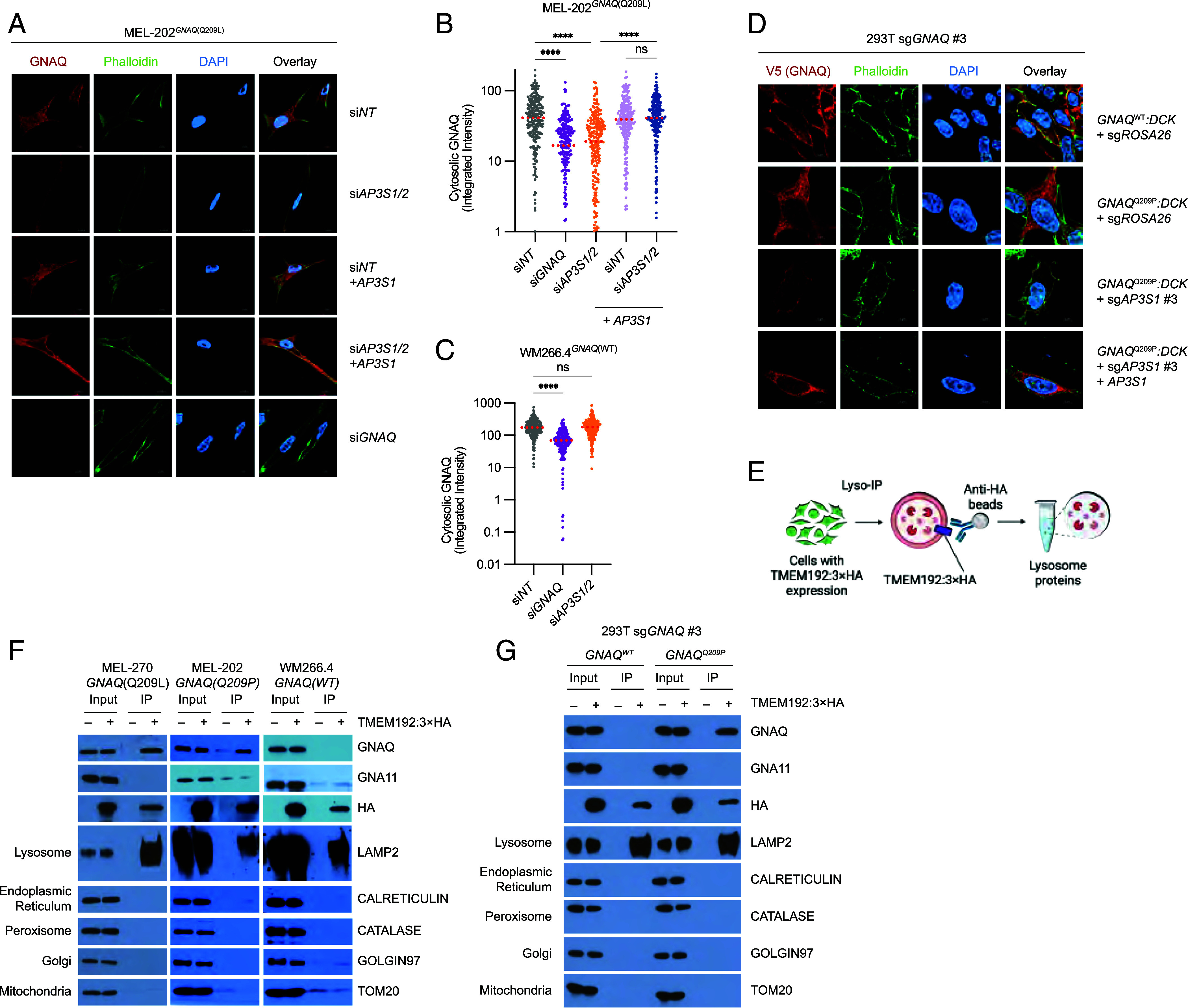
Constitutively active, but not unstimulated wild-type Gα_q_ localizes to the lysosomal compartment. (*A*) Immunofluorescence staining of endogenous Gα_q_ (red) in MEL-202*^GNAQ^*^(Q209L)^ cells transfected with the indicated siRNAs and/or reexpression of siRNA-resistant *AP3S1*. (Scale bar, 5 μm.) (*B*) Intensity of cytoplasmic Gα_q_ staining in MEL-202*^GNAQ^*^(Q209L)^ cells from (*A*). *n =* 231 cells. (*C*) Intensity of cytoplasmic Gα_q_^WT^ staining in WM266.4*^GNAQ^*^(WT)^ cells transfected with the indicated siRNAs. *n* = 210 cells. (*D*) Immunofluorescence staining in 293T cells expressing V5-tagged sg*GNAQ*#3 resistant *GNAQ^WT^:DCK* or sg*GNAQ*#3 resistant *GNAQ^Q209P^:DCK*, and/or reexpression of sgRNA-resistant *AP3S1*. (Scale bar, 5 μm.) (*E*) Schematic representation of Lyso-IP method workflow. (*F*) Immunoblot analysis of lysosomes purified (IP) by the Lyso-IP method in cells lacking (–) or expressing exogenous TMEM192:3×HA (+). (*G*) Immunoblot analysis of lysosomes purified (IP) by the Lyso-IP method from 293T sg*GNAQ*#3 cells expressing untagged sg*GNAQ*#3 resistant *GNAQ^WT^* or sg*GNAQ*#3 resistant *GNAQ^Q209P^* lacking (−) or expressing exogenous TMEM192:3×HA (+). Ordinary one-way ANOVA with multiple comparisons was used to determine statistical significance. ns, nonsignificant; *P < 0.05; ***P < 0.001; ****P < 0.0001. Error bars represent mean ± SEM.

Based on the observations above and previous studies (50, 67, 68), we hypothesized that AP-3σ regulates the trafficking of constitutively active Gα_q_ to the endolysosomal compartment and that loss of AP-3σ causes mislocalization and subsequent degradation of constitutively active Gα_q_. Accordingly, we evaluated the subcellular localization of wild-type and constitutively active Gα_q_ by immunofluorescence. Endogenous Gα_q_* in MEL-202*^GNAQ^*^(Q209L)^ and MEL-270*^GNAQ^*^(Q209P)^ localized mainly to the cytoplasm, whereas Gα_q_^WT^ in WM266.4*^GNAQ^*^(WT)^ cells localized to the plasma membrane (Fig. 3*A* and *SI Appendix*, Fig. S6 *A* and *B*). Knocking down of *AP3S1* and *AP3S2* decreased the intensity of cytoplasmic Gα_q_* staining in MEL-202*^GNAQ^*^(Q209L)^ and MEL-270*^GNAQ^*^(Q209P)^, which was restored by expressing *AP3S1* as determined by immunofluorescence and CellProfiler analysis (Fig. 3 *A* and *B* and *SI Appendix*, Fig. S6 *A* and *C*). Gα_q_^WT^ in WM266.4*^GNAQ^*^(WT)^ cells, mainly localized to the plasma membrane, and its protein level was not affected upon knockdown of *AP3S1*/*2* (Fig. 3*C* and *SI Appendix*, Fig. S6*B*).

To directly compare the localization of constitutively active Gα_q_ and unstimulated wild-type Gα_q_, we expressed V5-tagged sg*GNAQ*-resistant *GNAQ^Q209P^:DCK* or *GNAQ^WT^:DCK* in *GNAQ*-deficient 293T cells (Figs. 2*E* and 3*D*). Gα_q_*:DCK was mainly cytoplasmic, whereas wild-type Gα_q_ (Gα_q_^WT^:DCK) localized to the plasma membrane. *AP3S1* knockout decreased cytoplasmic Gα_q_*:DCK levels (Fig. 3*D* and *SI Appendix*, Fig. S6*D*). This phenotype was rescued by reexpressing *AP3S1*.

**Fig. 4.**
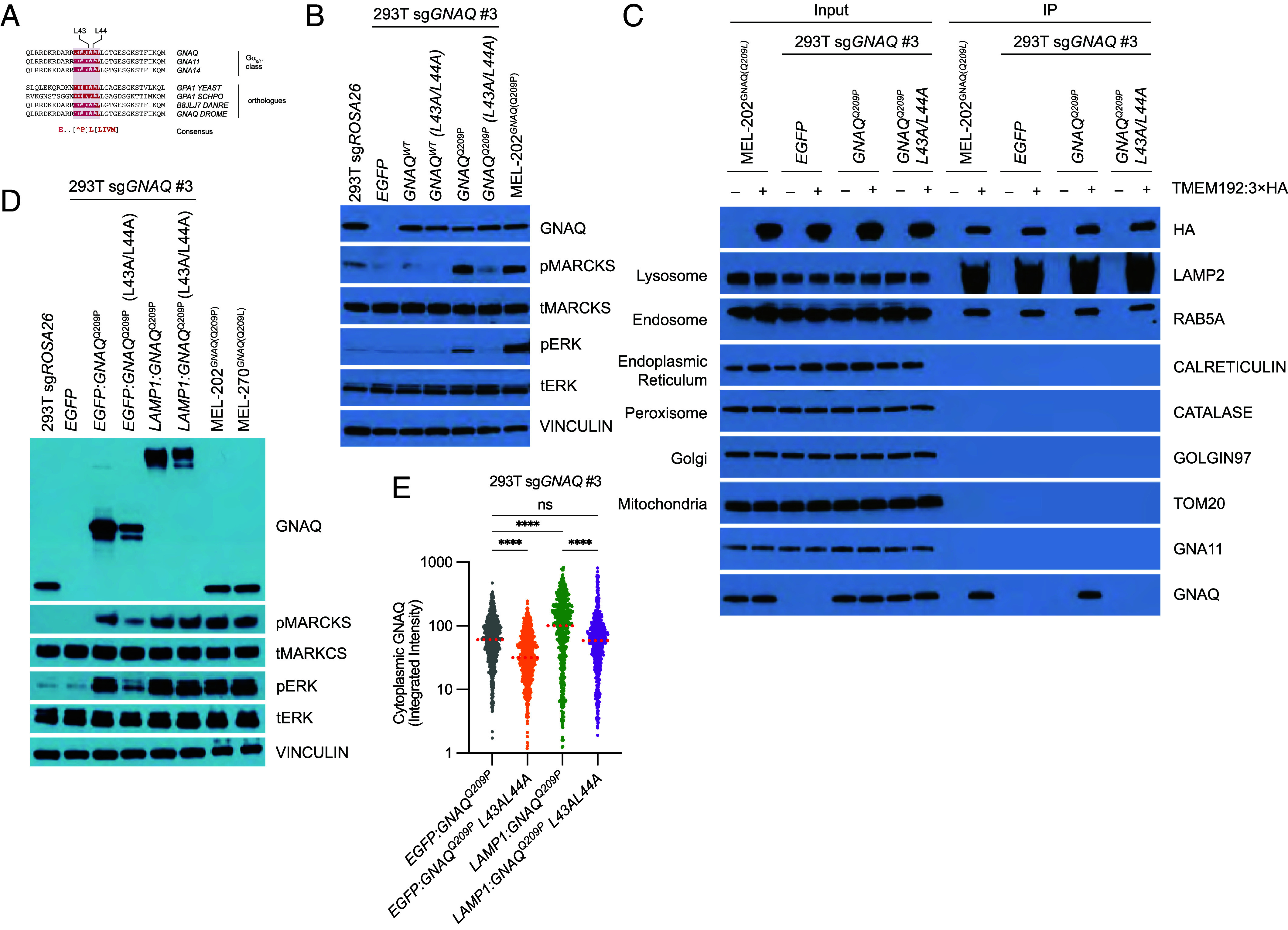
A conserved dileucine-based sorting signal is necessary for lysosomal localization and downstream signaling of constitutively active Gα_q_. (*A*) Protein sequence of Gα_q/11_ class of proteins in human, *Saccharomyces cerevisiae* (YEAST), *Schizosaccharomyces pombe* (SCHPO), *Danio rerio* (DNARE), and *Drosophila melanogaster* (DROME). The dileucine-based sorting signal (red). (*B*) Immunoblot of 293T lacking endogenous *GNAQ* (293T sg*GNAQ #*3) and transduced to express indicated cDNA. (*C*) Immunoblot analysis of lysosomes purified (IP) by the Lyso-IP method (D) in the indicated cells lacking (−) or expressing exogenous TMEM192:3×HA (+). (*D*) Immunoblot analysis in 293T lacking endogenous *GNAQ* transduced with the indicated cDNA. (*E*) The intensity of cytoplasmic Gα_q_ staining in cells from *SI Appendix*, Fig. S8*A*. *n* = 589 cells. All indicated exogenous *GNAQ* mutants and fusions are resistant to sg*GNAQ #*3. Ordinary one-way ANOVA with multiple comparisons was used to determine statistical significance. ns, nonsignificant; *P < 0.05; ***P < 0.001; ****P < 0.0001. Error bars represent mean ± SEM.

Since AP-3 regulates the trafficking of proteins to the endolysosomal compartment, we asked if Gα_q_* localized to lysosomes by immunopurifying lysosomes from cells expressing the lysosome-specific marker TMEM192 fused to three tandem HA epitopes (TMEM192:3×HA) (Lyso-IP) (Fig. 3*E*) (69). Confirming the specificity of this assay, immunoblot of immunoprecipitated lysosomes showed enrichment of lysosomal marker LAMP2 but not proteins restricted to other subcellular compartments. We found that Gα_q_ was enriched in the lysosomes immunopurified from uveal melanoma cell lines MEL-270*^GNAQ^*^(Q209P)^ and MEL-202*^GNAQ^*^(Q209L)^ and not in lysosomes immunopurified from WM266.4*^GNAQ^*^(WT)^ cells (Fig. 3*F*). To directly compare the localization of unstimulated wild-type and constitutively active Gα_q_, we performed Lyso-IP in Gα_q_-deficient 293T cells in which untagged sg*GNAQ*-resistant *GNAQ*^Q209P^ (Gα_q_*) and sg*GNAQ*-resistant *GNAQ*^WT^ (Gα_q_^WT^) were expressed at the same level as endogenous Gα_q_ (shown in Fig. 4*B*). As expected, Gα_q_* was enriched in the immunopurified lysosomes, whereas Gα_q_^WT^ was not (Fig. 3*G*).

To similarly test if Gα_q_ localizes to the endosomal compartment, we immunopurified intact endosomes from cells expressing the early endosome-associated protein, EEA1 fused to three tandem FLAG epitopes (3× FLAG:EEA1) (Endo-IP) (*SI Appendix*, Fig. S6*E*) (70). As expected, the endosomal marker RAB5A was enriched in the immunoprecipitated endosomes, whereas other subcellular compartment markers were not, thereby demonstrating the assay’s specificity. Gα_q_ was enriched in the endosomes immunopurified from MEL-202*^GNAQ^*^(Q209L)^ and not in endosomes immunopurified from SKMEL-28*^GNAQ^*^(WT)^ cells (*SI Appendix*, Fig. S6*F*). Finally, to test if Gα_q_* localizes to other lysosome-related organelles, melanosomes were immunopurified using the Melano-IP method (71) (*SI Appendix*, Fig. S6*E*). Despite enrichment of the melanosome marker Melan-a and the lysosomal marker LAMP2, Gα_q_* was not enriched in the melanosomes of the uveal melanoma cell line MEL-202*^GNAQ^*^(Q209L)^ (*SI Appendix*, Fig. S6*G*). Collectively, our data indicate that Gα_q_* localizes to endosomes and specific lysosomal-related organelles (endolysosomes) through an AP-3-dependent mechanism.

### A Conserved Dileucine-Based Sorting Signal Is Necessary for Endolysosomal Localization and Downstream Signaling of Constitutively Active Gα_q_.

AP-3 binds to its cargo proteins using sorting signals, one of which is a dileucine-based motif, [DE]XXXL[LI] (66, 72). We found that this dileucine motif is present in Gα_q_ and conserved among other mammalian Gα proteins (Fig. 4*A* and *SI Appendix*, Fig. S7*A*). Remarkably, this motif is also present in homologs in *Saccharomyces cerevisiae* (YEAST), *Schizosaccharomyces pombe* (SCHPO), zebrafish *Danio rerio* (DNARE), and *Drosophila melanogaster* (DROME) (Fig. 4*A* and *SI Appendix*, Fig. S7*A*).To evaluate if this dileucine motif is necessary for Gα_q_-mediated signaling, we stably expressed sg*GNAQ*-resistant wild-type or constitutively active forms of Gα_q_ in which the dileucine residues were mutated to alanine (L43A/L44A) in 293T cells lacking endogenous Gα_q_^WT^. To minimize artifacts, these proteins were expressed at near-endogenous levels without any epitope tags, and the expression level of all exogenous Gα_q_ proteins was equalized by titrating the lentivirus expressing these GNAQ variants (Fig. 4*B*). Immunoblot analysis showed that mutating the dileucine motif of Gα_q_* (Gα_q_*^, L43A/L44A^) reduced pERK and pMARCKS levels compared to cells expressing Gα_q_*, while the levels of pERK and pMARCKS in Gα_q_^WT^ and Gα_q_^WT, L43A/L44A^ expressing cells were comparable (Fig. 4*B*). We considered the possibility that impaired signaling by the dileucine mutant could reflect diminished binding to downstream effector or regulator proteins that associate with active Gα_q_. Therefore, we evaluated whether these mutant proteins could interact with the regulator GAIP or the effector GRK2 using GST pulldown assays (73). However, we found that mutation of Gα_q_* at L43/L44 did not affect its ability to bind GAIP or GRK2 (*SI Appendix*, Fig. S7 *B* and *C*). To test the effect of mutating the dileucine motif on lysosomal localization of Gα_q_ in these cell lines, we additionally performed Lyso-IP. Immunoblot of isolated lysosomes showed that mutation of the dileucine motif prevented lysosomal localization of Gα_q_* (Fig. 4*C*).

Based on these results, we reasoned that impaired signaling in the dileucine mutant was due to failure of AP-3σ-dependent trafficking of Gα_q_* to the endolysosomal compartment. To test this hypothesis, we fused the lysosomal-associated membrane protein 1, LAMP1, to the N terminus of Gα_q_* and Gα_q_*^, L43A/L44A^. Corresponding *EGFP* fusions were also generated as controls. Immunofluorescence showed that the expression pattern of LAMP1:Gα_q_* and LAMP1:Gα_q_*^, L43A/L44A^ were similar, and resembled the expression of EGFP:LAMP1 (*SI Appendix*, Fig. S8*A*). Lysosomal localization of the LAMP1 fusion proteins was verified by Lyso-IP (*SI Appendix*, Fig. S8*B*). Immunoblot, immunofluorescence, and CellProfiler analysis showed that expression levels of EGFP and LAMP1 fused Gα_q_*^, L43A/L44A^ were less than those of EGFP and LAMP1:Gα_q_*fusion proteins (Fig. 4 *D* and *E* and *SI Appendix*, Fig. S8*A*). Despite the relatively lower expression, MAPK and PKC signaling in LAMP1:Gα_q_*^, L43A/L44A^ expressing cells was comparable to LAMP1:Gα_q_* expressing cells and higher than EGFP:Gα_q_*^, L43A/L44A^ expressing cells (Fig. 4*D*). This difference in signaling was not due to differences in *LAMP1:GNAQ*^Q209P, L43A/L44A^ and *EGFP:GNAQ*^Q209P, L43A/L44A^ mRNA levels, as RT-qPCR analysis and found no major difference (*SI Appendix*, Fig. S8*C*). Thus, restoring the lysosomal localization of the Gα_q_*^, L43A/L44A^ dileucine mutants is sufficient to rescue their downstream signaling. Collectively, our data show that the conserved dileucine-based endolysosomal sorting motif in Gα_q_* is necessary for Gα_q_*-dependent signaling.

### A Conserved Dileucine-Based Sorting Signal Is Necessary for Constitutively Active Gα_q_-Mediated Growth of Uveal Melanoma Cells.

Based on the impact of the dileucine mutation on the subcellular localization, and downstream signaling of Gα_q_*, we next evaluated if AP-3σ or the dileucine residues were required for cell growth in a cell line that is dependent on constitutively active Gα_q_. Knocking out *GNAQ* or *AP3S1/2* impaired the growth of a uveal melanoma cell line, MEL-202*^GNAQ^*^(Q209L)^ as determined by a FACS-based competition assay (*SI Appendix*, Fig. S9*A*). The growth impairment phenotype seen in the *AP3S1/2* knockout MEL-202*^GNAQ^*^(Q209L)^ cells was rescued by expressing the sgRNA-resistant *AP3S1* cDNA.

**Fig. 5.**
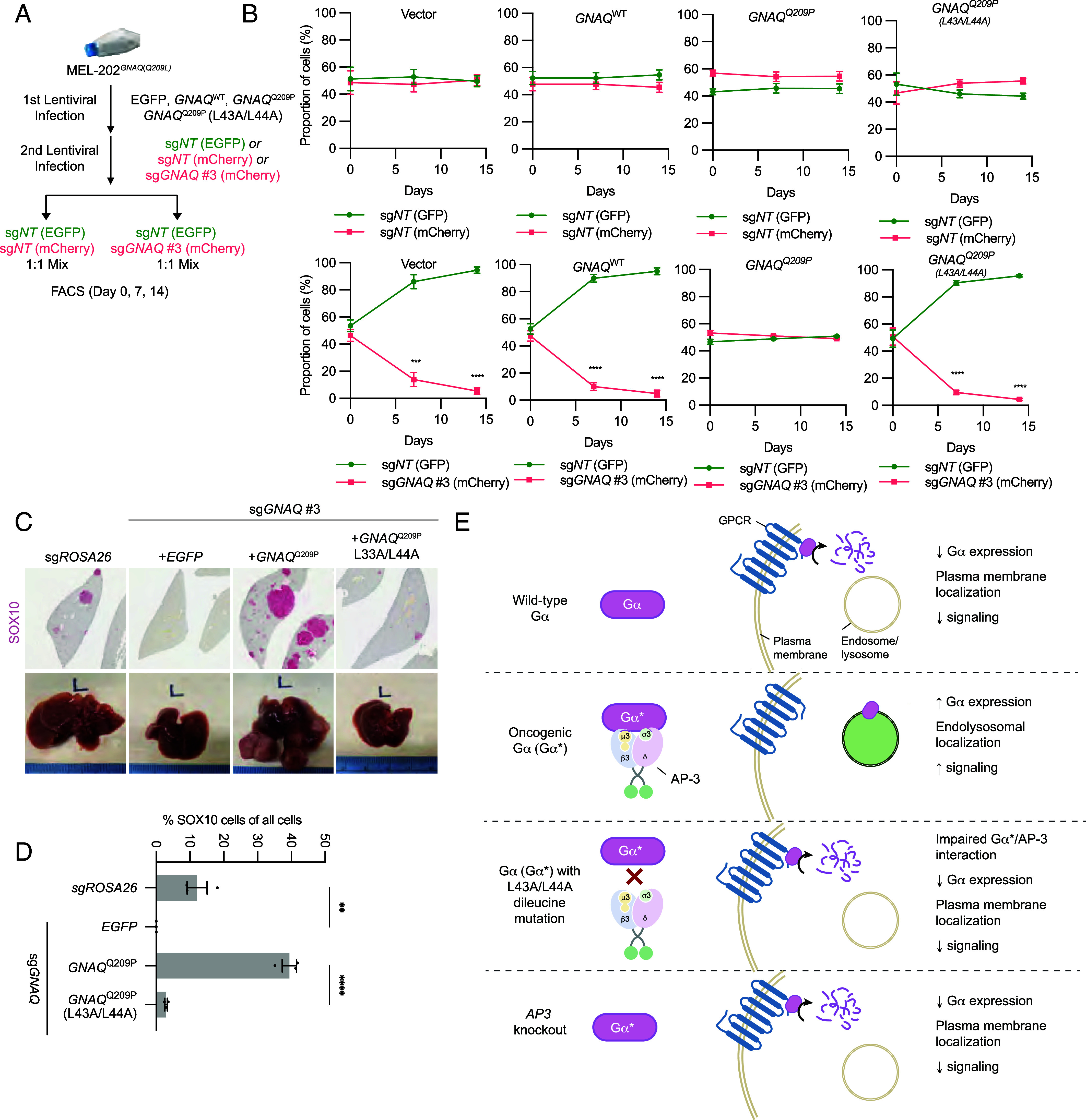
A conserved dileucine-based sorting signal is necessary for constitutively active Gα_q_-mediated growth of uveal melanoma cells (*A*) Schematic representation of FACS competition assay. (*B*) Percentage of EGFP and mCherry positive cells as determined by the FACS competition assay. (*C*) Representative images of whole livers and SOX10-stained sections of the livers harvested from three mice injected with the indicated cell lines, 10 wk postinjection. (*D*) Quantification of SOX10 positive cells in the livers of mice from (*C*). (*E*) Proposed mechanism of AP-3σ/dileucine motif regulation of Gα* localization and signaling: Gα^WT^ mainly localizes to the cytoplasmic face of the plasma membrane and undergoes recycling post–GPCR activation. AP-3σ binds to the dileucine motif on constitutively active/oncogenic Gα (Gα*) and traffics it to the endolysosomal compartment, where it mediates sustained signaling. Loss of AP-3σ or mutation in the dileucine motif impairs AP-3σ-Gα* interaction, resulting in mislocalization and degradation of Gα* and, consequently, downregulation of signaling. Two-way ANOVA with multiple comparisons was used to determine statistical significance for (*B*) and ordinary one-way ANOVA with multiple comparisons was used to determine statistical significance for (*D*). ns, nonsignificant; *P < 0.05; ***P < 0.001; ****P < 0.0001. Error bars represent mean ± SEM.

To directly evaluate the role of the dileucine motif in Gα_q_*-mediated growth, we stably expressed *GNAQ*^Q209P^, *GNAQ*^Q209P, L43A/L44A^, or *GNAQ*^WT^ in MEL-202*^GNAQ^*^(Q209L)^ cells. Next, we knocked out endogenous *GNAQ* and evaluated *if GNAQ*^Q209P^ *GNAQ*^Q209P, L43AL44A^ or *GNAQ*^WT^ could rescue cell growth using a FACS-based competition assay (Fig. 5*A*). Expression of Gα_q_ was similar across all cell lines (*SI Appendix*, Fig. S9*B*). *GNAQ*^Q209P^ rescued the growth defect phenotype caused by knocking out endogenous *GNAQ*, whereas *GNAQ*^WT^ and *GNAQ*^Q209P, L43AL44A^ could not (Fig. 5*B*).

Next, we evaluated the requirement of the dileucine motif in Gα_q_*-mediated growth and metastasis in a patient-relevant murine model. MEL-202*^GNAQ^*^(Q209L)^ cells stably expressing sg*GNAQ*-resistant *GNAQ*^Q209P^, *GNAQ*^Q209P, L43A/L44A^, or *EGFP* were transduced with lentivirus expressing sg*GNAQ* or sg*ROSA26* (control sgRNA). Each cell line was injected intravenously into the tail vein of 3, 8-wk-old NSG mice. In this model, macrometastasis is observed in the liver, mimicking observations of most patients (74, 75). After 10 wk, we harvested livers and evaluated for the presence of uveal melanoma cells using SOX10 immunohistochemistry. MEL-202*^GNAQ^*^(Q209L)^ cells expressing sg*GNAQ*-resistant *GNAQ*^Q209P^ and sg*GNAQ* formed prominent SOX10-positive tumors. As expected, MEL-202*^GNAQ^*^(Q209L)^ cells expressing the control sgRNA also formed SOX10-positive tumors. In comparison, MEL-202*^GNAQ^*^(Q209L)^ cells expressing sg*GNAQ*-resistant *GNAQ*^Q209P, L43A/L44A^ and sg*GNAQ* formed fewer and smaller SOX10-positive tumors while the MEL-202*^GNAQ^*^(Q209L)^ cells expressing a control cDNA (*EGFP*) and sg*GNAQ* did not form any tumors (Fig. 5 *C* and *D*). Together, our data show that the dileucine motif is necessary for Gα_q_*-mediated growth of cells that are dependent on constitutively active Gα_q_.

## Discussion

In this study, we identify regulators of Gα_q_* through a genome-wide, positive selection screen. Analysis of these data indicates a prominent role of endolysosomal localization in regulating Gα_q_* abundance and signaling. We show that, unlike Gα_q_^WT^, which mainly localizes to the plasma membrane, Gα_q_* primarily localizes to the cytoplasm, specifically in endosomes and lysosomes. We found that the trafficking protein AP-3 is necessary for the endolysosomal localization and signaling of Gα_q_*. Further, we found that Gα_q_* contains an AP-3 cargo recognition motif (dileucine-based sorting signal) that is essential for its subcellular localization and signaling. Mutation of the recognition motif also compromises the growth of uveal melanoma cells that depend on constitutive Gα_q_ signaling. Together, these observations suggest a mechanism by which G-protein signaling is compartmentalized and indicate an important functional role for Gα_q_* endolysosomal localization.

Numerous studies have previously demonstrated the localization of Gα family members to endosomes (34, 37–39) and lysosomes (76). However, the molecular mechanisms driving the endolysosomal localization and the functional consequences of endolysosomal localization have been unclear. The functional relevance of G proteins in endosomal GPCR signaling has also been difficult to definitively discern, as approaches that activate GPCR signaling may lead to the activation of multiple families of G proteins (reviewed in ref. 18). Our approach of using a constitutively active mutant version of Gα_q_ overcomes some of these difficulties in interpreting the role of G proteins in endosomal GPCR signaling in studies where GPCR signaling is activated via receptor agonists.

Lysosomes have long been considered sites of degradation, but numerous studies have demonstrated that they can also serve as sites of vital signaling pathways. For example, mTOR localizes in its active form to the surface of lysosomes (77). Thus, it is intriguing to hypothesize that this subcellular localization of active Gα_q_ may lead to the activation of specific effectors localized to the endolysosomal compartment. In the yeast *Saccharomyces cerevisiae*, the constitutively active mutant of Gα, Gpa1^Q323L^, was found to be present at endosomes rather than the plasma membrane (78). Endosomal Gpa1^Q323L^ regulated some, but not all, mating switching phenotypes through interaction with the endosomally localized phosphatidylinositol 3-kinase Vps34.

The reasons why endolysosomal localization is required for Gα_q_* signaling and growth remain to be clarified. It has been suggested that endosomal GPCR signaling may be protected from its downregulation by β-arrestins specifically in this compartment. Alternatively, the endosomal compartment itself may drive specific signaling pathways (79, 80). Based on these findings and our observations, it is tempting to speculate that distinct downstream effectors of GPCR signaling are compartmentalized similarly in mammalian cells. Constitutively activated Gα_q_ can activate PKC isoforms (57, 81), some of which are localized to the lysosomal compartment (82). The myristoylated alanine-rich C kinase substrate (MARCKS), a prominent substrate for PKC, is known to translocate to the lysosome upon phosphorylation by PKC, where it is protected from degradation by the lysosomal enzyme, cathepsin B (83, 84). Thus, the lysosome could serve as a site for some but not necessarily all Gα_q_*-mediated signaling. If distinct subcellular compartments of Gα_q_* are linked to specific functions, it could create the opportunity to target particular Gα_q_* effector pathways, for example, by inhibiting the interaction of Gα_q_* with AP-3. Irannejad et al. developed a chemical method to acutely squelch G-protein signaling at specific membrane locations (27), thus creating the possibility of functional selectivity of GPCR-directed drug action.

Our study identifies a dileucine-based sorting signal within Gα_q_ that is necessary for the lysosomal localization of constitutively active Gα_q_*. Mutation of this motif did not result in loss of ability to bind to effector and regulator proteins. Further, the activity of Gα_q_* lacking the AP-3 binding motif (dileucine-based sorting signal/dileucine motif) can be rescued by relocalizing to the lysosome. These observations suggest that the dileucine motif, while necessary for its localization, is not directly essential for its constitutive activity. We have also demonstrated the necessity of the dileucine motif for Gα_q_*-mediated growth of uveal melanoma cells that are dependent on Gα_q_* for survival in PDX models. However, we cannot formally rule out the possibility that additional adapter proteins bind to the dileucine motif. In addition to AP-3, AP-2 also recognizes and binds to dileucine-based sorting signals in its cargo proteins through its α-σ2 hemicomplex (38, 85, 86). AP-3 and AP-2 share similar binding site residues but are known to function at different subcellular locations (87). While AP-2 is mainly involved in endocytosis from the plasma membrane (88), AP-3 is present at endosomes and the trans-Golgi network and is predominantly involved in trafficking to endosomes, lysosomes, and lysosome-related organelles (89). Although our screen did not identify members of AP-2, it is possible that Gα_q_* may be trafficked through the same motif to different subcellular compartments. Furthermore, we hypothesize that conformational changes caused by physiological activation could expose the dileucine motif in Gα^WT^ to adapter proteins that recognize and bind to dileucine-based sorting signals. However, given the transient nature of the Gα signaling under physiologic conditions, additional models and assays will be required to formally address this in the future. Our study focuses on Gα_q_, but the AP-3 cargo recognition motif was conserved across most Gα proteins in humans and homologs from yeast to zebrafish. Further studies will be necessary to determine the specific roles of AP-3 in other G proteins and across various species.

We have shown that loss of AP-3σ results in a decrease in cytoplasmic Gα_q_*. Studies on fibroblasts from Hermansky-Pudlak syndrome (HPS) patients showed that mutations in the AP-3 β3A subunit, which in turn affects the stability of σ3, result in altered trafficking of lysosomal membrane proteins CD63, LAMP1, and LAMP2 to the cell surface (50, 68). Mislocalized proteins are often degraded (50, 68). This further suggests that the decrease in abundance of Gα_q_* we observe in the absence of AP-3σ could be a consequence of altered trafficking of Gα_q_* to the cell surface followed by degradation (68) (Fig. 5*E*).

In summary, we performed a genome-scale positive-selection screen that identified AP-3 as a regulator of constitutively active Gα_q_ signaling. Given that AP-3 does not regulate the abundance of wild-type Gα_q_ or growth signaling in wild-type Gα_q_ expressing cells and that distinct pathways may be regulated by endolysosomal Gα_q_*, our findings may provide a basis for therapeutically targeting location-specific aspects of Gα_q_* function selectively. Potential for translation of these findings to uveal melanoma patients could involve blocking the interaction between AP-3 and the dileucine motif using small molecules, or inactivation of endolysosomal trafficking complexes, some of which are dependent on enzymatic activity. Thus, targeting the trafficking of specific subcellular pools of constitutively active Gα proteins could be exploited to develop therapies in various conditions that exhibit constitutively active G-protein signaling.

## Materials and Methods

All experimental details are provided in the *SI Appendix*.

## Supplementary Material

Appendix 01 (PDF)

Dataset S01 (XLSX)

Dataset S02 (XLSX)

Dataset S03 (XLSX)

Dataset S04 (XLSX)

## Data Availability

All study data are included in the article and/or supporting information.
