# Supplementary material for: Oncogenic Gα signaling requires AP-3-dependent recruitment to the endolysosomal compartment
Source: Proc Natl Acad Sci U S A. 2026 Jul 22;123(30):e2615774123. doi: 10.1073/pnas.2615774123 (PMC13415926; doi:10.1073/pnas.2615774123)
Supplement: Supplementary file 1 — Appendix 01 (PDF) [file pnas.2615774123.sapp.pdf]

PNAS

Supporting Information for
Oncogenic Gα Signaling Requires AP-3-Dependent Recruitment to the Endo-lysosomal Compartment

Megha Shettigar[1], Salomé Moulière[1], Larissa Isenegger[1], Alexander L. DeVine[1], Cécile Gstalder[1], Vidyasagar Koduri[2], John G. Doench[3], Bruce Ksander[4], Mikel Garcia-Marcos[5], William G. Kaelin Jr*[1,2], Rizwan Haq*[1,2]

*Co-corresponding authors:
Rizwan Haq, MD PhD. **Email:** rizwan_haq@dfci.harvard.edu
William G. Kaelin, Jr. **Email**: william_Kaelin@dfci.harvard.edu

This PDF file includes:

Supporting text
Figures S1 to S9
Legends for Datasets S1 to S4
SI References

Other supporting materials for this manuscript include the following:

Datasets S1 to S4

Supporting Information Text

Supplementary Materials and Methods

Cell lines and cell culture

293T and SKMEL-28$^{\mathit{GNAQ}(\mathrm{WT})}$ cells were cultured in DMEM, supplemented with 5% and 10% FBS, respectively, and 1% penicillin-streptomycin. MEL-202$^{\mathit{GNAQ}(\mathrm{Q209L})}$, MEL-270$^{\mathit{GNAQ}(\mathrm{Q209P})}$, MP41$^{\mathit{GNA11}(\mathrm{Q209L})}$, and WM266.4$^{\mathit{GNAQ}(\mathrm{WT})}$ cells were cultured in RPMI, supplemented with 10% FBS and 1% penicillin-streptomycin. For Fig. S1B, the serum-starved cells were cultured in the corresponding media lacking serum for 24 hours. Cell lines were maintained at 37°C in 5% CO_2. Cell lines were tested biweekly for mycoplasma using PCR-based screening (PCR Mycoplasma Detection Kit, Cat# G238, Applied Biological Materials Inc.) and verified to be mycoplasma negative. All cell lines were authenticated using the ATCC Sample Collection Kit Cell Authentication Service.

Generation of knockout cell lines using CRISPR/Cas9 targeting

All knockouts were generated using CRISPR/Cas9-based gene editing. The oligonucleotides purchased from Eton Biosciences (Dataset S3) were annealed and cloned into lentiCRISPR or LentiGuide plasmids. The generated plasmids were co-transfected with PAX2 and pMD2.G viral packaging plasmids into HEK 293T Lenti-X cells (Clontech) using TransIT-LT1 (Mirus Bio LLC). Cells were infected with lentivirus harvested after two days and placed under drug selection using puromycin (1 µg/mL, InVivoGen) for 48 hours or Zeocin (250 µg/mL, InVivoGen) for 14 days. Cells were allowed to edit for two weeks post-viral infection, after which they were validated by western blot. Knockout efficacy was determined by Sanger sequencing and the TIDE analysis software[1].

Cloning and mutagenesis

pDONR plasmids encoding *GNAQ* cDNA resistant to sg*GNAQ* #3, Q209P mutant, L43A/L44A mutant, *AP3S1,* and *AP3S2* cDNA resistant to sg*AP3S1* #3, site-directed mutagenesis was performed using In-Fusion HD Cloning Kit (Takara Bio) and oligos purchased from Eton Biosciences (Dataset S3). Gateway cloning was then used to introduce the cDNA into pLX304-gateway-DCK-IRES-EGFP to generate pLX304-GNAQ Q209P-DCK-IRES-EGFP, pLX304-GNAQ Q209P sgRES-DCK-IRES-EGFP and pLX304-GNAQ WT sgRES-DCK-IRES-EGFP or into pLenti-PGK-Hygromycin-DEST to generate pLenti-PGK-GNAQ WT sgRES, pLenti-PGK-GNAQ WT L43A/L44A sgRES, pLenti-PGK-GNAQ Q209P sgRES, pLenti-PGK-GNAQ Q209P L43A/L44A sgRES, pLenti-PGK-AP3S1 sgRES and pLenti-PGK-AP3S2 sgRES expression plasmids.

LAMP1:GNAQ$^{\mathrm{Q209P}}$/*LAMP1:GNAQ*$^{\mathrm{Q209P,\ L43A/L44A}}$ fusions were generated by PCR-based linearization of pLVX-EF1a-LAMP-1-mGFP-IRES-Puromycin to exclude mGFP using (*LAMP1* vector linearization F, R primers) (see Dataset S3). *GNAQ*$^{\mathrm{Q209P}}$/ *GNAQ*$^{\mathrm{Q209P,\ L43A/L44A}}$ was amplified from pDONR GNAQ Q209P sgRES CLOSED or pDONR-GNAQ Q209P L43A/L44A sgRES CLOSED plasmids using primers *LAMP1 GNAQ* INSERT F, R (see Dataset S3) and cloned into the linearized plasmid using In-Fusion cloning. pDONR plasmids expressing this fusion were generated by TOPO cloning (pENTR/D-TOPO Cloning Kit, Clontech) using primers TOPO *LAMP1 GNAQ* F, R (see Dataset S3). Gateway cloning was then used to introduce the cDNA into pLenti-PGK-Hygromycin-DEST to generate pLenti-PGK-LAMP1-GNAQ Q209P sgRES and pLenti-PGK-LAMP1-GNAQ Q209P L43A/L44A sgRES expression plasmids.
pDONR plasmid expressing *EGFP:GNAQ*$^{\mathrm{Q209P}}$/*EGFP:GNAQ*$^{\mathrm{Q209P,\ L43A/L44A}}$ fusion was generated by linearizing the pDONR-LAMP1-GNAQ-Q209P-sgRES-STOP plasmid using primers LV F,R (see Dataset S3). EGFP was amplified using primers LI F, R (see Dataset S3) from pLVX-EF1a-EGFP-VAMP5-IRES-Puromycin and cloned into the linearized plasmid using In-Fusion cloning (Clontech). All plasmids generated were verified by Sanger sequencing before being used. Gateway cloning was then used to introduce the cDNA into pLenti-PGK-Hygromycin-DEST to generate pLenti-PGK-EGFP-GNAQ Q209P sgRES and pLenti-PGK-EGFP-GNAQ Q209P L43A/L44A sgRES expression plasmids.

siRNA
MEL-202$^{GNAQ(Q209L)}$, MEL-270$^{GNAQ(Q209P)}$, MP41$^{GNA11(Q209L)}$, and WM266.4$^{GNAQ(WT)}$ cells were seeded in 6 well plates at 3×10^4 cells/mL in 6 well plates. After 24 hours, siRNA duplexes (30 picomoles per well) were transfected into the cells using Lipofectamine RNAiMAX (Invitrogen) using the protocol provided by the manufacturer. After 72 hours, the cells were processed for immunofluorescence experiments and immunoblot analysis.

Immunoblot analysis
Whole-cell lysates were generated using RIPA lysis buffer (Boston Bioproducts) supplemented with cOmplete Mini protease inhibitor (Roche) and Phospho-STOP phosphatase inhibitor (Roche). For Fig. S1B, after lysing cells in RIPA, the cells were briefly sonicated on ice. Protein concentration was determined using the BCA Protein Assay Kit (Thermo Scientific). Samples were denatured with reducing Laemmli buffer at 95°C for 5 min. Samples were resolved on 4-20% Criterion TGX Stain-Free Precast Gels (BioRad) at 150 V for 1 hour. Proteins were transferred to a 0.2µm TransBlot Turbo Midi size nitrocellulose membrane using Trans-Blot Turbo (BioRad). The membranes were blocked for one hour at room temperature (RT) in 5% milk or 5% BSA (before probing for pMARCKS and pRASGRP3) in 1× TBST (Tris-buffered saline with Tween 20), washed for 10 min in 1× TBST, and then incubated with primary antibodies (details in Dataset S4) in 5% BSA (Bovine Serum Albumin) in 1× TBST at 4 °C overnight. Membranes were washed thrice for 10 minutes and incubated with horseradish peroxidase (HRP)-conjugated secondary antibodies in 5% milk in 1× TBST for 1 hour at RT. Membranes were washed three times for 10 minutes. Antibodies bound to the membrane were detected using chemiluminescent immunoblotting detection reagents: Pierce ECL Western Blotting Substrate (Thermo Scientific Cat#32106), SuperSignal West Dura Extended Duration Substrate (Thermo Scientific Cat# 34076), SuperSignal West Femto Maximum Sensitivity Substrate (Thermo Scientific Cat#34096). Films were developed in a darkroom using a Kodak X-OMAT 2000A processor.

CRISPR screen
293T cells were infected with lentivirus expressing pLX304-GNAQ Q209P-DCK-IRES-EGFP and pLX304-DCK-IRES-EGFP. 24 hours later, the cells were selected with blasticidin (10 µg/mL, InVivoGen) for 5 days. Protein expression was tested by immunoblot analysis. On day 1 of the screen, 2×10^8 293T *DCK* and 293T $GNAQ^{Q209P}$*:DCK* cells (approximately 800 cells/sgRNA, MOI 0.3-0.5) were seeded for infection with the whole genome Brunello library (76441 sgRNAs). The cells were seeded in 30, 15 cm plates at 7.1×10^6 cells per plate in DMEM supplemented with 5% FBS, 1% penicillin, and streptomycin (whole media). On day 2, the media was replaced with 25 mL of DMEM containing 10% FBS, 8µg/mL polybrene, and 2.2 mL of whole genome Brunello library (CP0043) lentivirus expressing Cas9. On day 3, the media was replaced with whole media. On day 4, cells were placed under puromycin selection (1 µg/mL) in whole media for 48 hours. After 48 hours, the media was replaced with whole media, and the cells were allowed to edit for 14 days. At the end of 14 days, cells were seeded in 3 T225 flasks per cell line, at 20×10^6 cells per flask in whole media containing 12 µM BVdU. Eighty million cells were harvested, washed with PBS, pelleted, and frozen for genomic DNA isolation (time point T0). Cells were treated with BVdU for 28 days. Eighty million cells were harvested, washed with PBS, pelleted, and frozen for genomic DNA isolation at 14(T14), 21(T21), and 28(T28) days. Genomic DNA was isolated using the NucleoSpin Blood XL kit (Macherey-Nagel, Cat# 740950.10) according to the protocol provided by the manufacturer and sent to the Broad Institute of Harvard and MIT for next-generation sequencing. Data of raw Illumina reads normalized between samples using $\log2[(\text{sgRNA reads/total reads for sample}) \times 10^6 + 1]$. The relative enrichment of each individual sgRNA after BVdU treatment was determined by subtracting the T0 data from the end time point after BVdU selection (T14/T21/T28). Hypergeometric analysis of this data was performed using the Broad Institute GPP Web Portal (https://portals.broadinstitute.org/gpp/public/).

GST Pulldown
MEL-270$^{GNAQ(Q209P)}$, 293T sg*GNAQ* #3 cells expressing *EGFP*, $GNAQ^{WT}$, $GNAQ^{WT,\ L43A/L44A}$, $GNAQ^{Q209P,}$ and $GNAQ^{Q209P,\ L43A/L44A}$ were seeded in 10cm dishes and grown to 70% confluence. Cells were washed with 10 mL 1X PBS and harvested by scraping in 1 mL of 1× PBS. Cells were

centrifuged at 550 × g for 5 minutes at 4°C. Cells were lysed in 240 µL of lysis buffer [20 mM Hepes, pH 7.2, 125 mM K(CH3COO), 5mM Magnesium acetate, 0.4% (v/v) Triton X-100, 10 mM β-glycerophosphate, 1 mM DTT, 30 µM GDP, phosphatase and protease inhibitors] and incubated on ice for 10 minutes. Lysate was centrifuged at 14,000 × g for 10 minutes at 4°C. 240 µl of supernatant was transferred to a clean tube on ice. Protein concentration was measured by the BCA assay. 10 µL of lysate was reserved as input. 5 µg of GST, GST-fused proteins per sample (GST-GAIP and GST-GRK2^{RH}) were immobilized on GSH-agarose beads by incubation in 1mL of binding buffer [50 mM Tris-HCl, pH 7.4, 100 mM NaCl, 0.4% (v/v) NP-40, 5 mM EDTA, 30 µM GDP, 2 mM DTT, protease and phosphatase inhibitors] at room temperature for 90 minutes with gentle rotation. Beads were washed thrice in 1ml of binding buffer, centrifuged at 10,000 × g for 30 seconds at 4°C, the supernatant was discarded, and the beads were resuspended in binding buffer. These beads were then added to the cell lysates (approximately 400 µg of total protein) and incubated for 4h at 4°C with constant rotation. Beads were washed 3 times with 1mL of PBST wash buffer (1× PBS, 0.1% Tween-20, 10mM $MgCl_2$, 5mM EDTA, 30 µM GDP, 1 mM DTT). Beads were centrifuged at 10,000 × g for 30 seconds at 4°C. Proteins bound to the beds were eluted once with 10 µl of reducing 4× Laemmli buffer, incubated at 65°C for 10 minutes, spun down, and the supernatant was transferred to a fresh tube on ice. The second elution was performed similarly, and the eluates were pooled. GDP, DTT, protease inhibitors, and phosphatase inhibitors were added to buffers right before use. Purified GST, GST-GAIP, GST-GRK2^{RH} were provided by Garcia-Marcos Lab[2].

Lysosomal, endosomal, and melanosomal immunoprecipitation
Cells were infected with lentivirus expressing pLJC5-Tmem192-3xHA, pHAGE-3xFLAG-EEA1, and pLJC5-GPR143-mScarlet-3×HA. After 24 hours, cells were selected with puromycin (1 µg/mL, InVivoGen) for 48 hours. Protein expression was tested by immunoblot analysis. Cells stably expressing these plasmids and control cells not expressing these plasmids from 2, 70-80% confluent 15 cm plates were used for the assays. Cells were washed with ice-cold PBS and then harvested by scraping in 1 mL of KPBS (25 mM KCl, 100 mM KH2PO4, pH 7.25, adjusted with 1M KOH). Cells were centrifuged at 1000× g for 2 minutes at 4°C. The cell pellet was then resuspended in 1 mL of KBPS buffer supplemented with protease and phosphatase inhibitors (complete buffer) and lysed using a Dounce homogenizer on ice. The lysed cells were centrifuged at 1000 × g for 2 minutes at 4°C. The protein concentration of the supernatant was determined, and 10 µL of the supernatant was reserved as input. Homogenate equivalent to 1000 µg of protein was added to the KBPS complete buffer to make up the volume to 1 mL and incubated with 60 µL of anti-HA/FLAG magnetic bead slurry (washed 3 times with KPBS) at 4 °C for 50 minutes with gentle rotation. Beads were separated from the homogenate using a magnetic stand. For Lyso-IP and Melano-IP, post-incubation, the beads were gently washed 3 times with KBPS complete buffer supplemented with 500 mM NaCl, followed by a single wash with KBPS complete buffer. For Endo-IP, the beads were gently washed twice with KBPS complete buffer supplemented with 150 mM NaCl and once with KBPS complete buffer. Immunoprecipitated proteins were eluted by incubating beads in 1× Triton-X lysis buffer supplemented with protease and phosphatase inhibitors for 30 min at 4 °C with gentle rotation. Protein samples were denatured for SDS-PAGE by incubating with Laemmli buffer with reducing agent β-mercaptoethanol at 95 °C for 5 min.

Immunofluorescence
No.1.5 cover glasses coated with 0.1 % (w/v) poly-L-lysine solution in water were used. Cells were seeded on the cover glass in 6-well plates. Coverslips were washed with 1× PBS supplemented with calcium chloride and magnesium chloride (IF buffer). Cells were then fixed in 4% paraformaldehyde diluted in IF buffer for 10 minutes at room temperature (RT). Coverslips were washed thrice with IF buffer. For experiments performed to visualize YAP, cells were permeabilized with 0.3% Triton X-100 in IF buffer for 10 minutes at RT. For experiments performed to visualize endogenous GNAQ or V5-tagged proteins, cells were permeabilized and blocked with 0.1% saponin in saponin-blocking buffer (IF buffer supplemented with 10% goat serum, 0.3 M glycine, and 0.1% saponin) for 1 hour at RT and then stained with anti-GNAQ rabbit monoclonal primary antibody (1:500) or anti-V5 tag mouse monoclonal antibody (1:2000) in

saponin blocking buffer. Coverslips were washed thrice with saponin wash buffer (0.01% saponin in IF buffer). For YAP staining, cells were blocked in Triton X-100 blocking buffer (IF buffer supplemented with 10% goat serum, 0.3 M glycine, and 0.05% Triton X-100) for an hour at RT and then stained with anti-YAP rabbit monoclonal primary antibody (1:400) in blocking buffer for an hour at RT. Coverslips were washed thrice with Triton X-100 wash buffer (0.01% Triton X-100 in IF buffer) or saponin wash buffer (0.01% saponin in IF buffer). Cells were stained with goat anti-rabbit/mouse Alexa Fluor 647 secondary antibody (1:2000) in saponin or TritonX-100 blocking buffer for 1 hour at RT. Coverslips were washed with Triton X-100 or saponin wash buffer once and then stained with phalloidin per the manufacturer's instructions for 30 minutes at RT. Coverslips were washed thrice with Triton X-100 or saponin wash buffer. Coverslips were mounted in ProLong Diamond Antifade mounting medium containing DAPI and imaged at 63× magnification using a Zeiss LSM 980 confocal microscope for all $G\alpha_q$ localization figures or at 63× magnification using a Leica Thunder microscope for all YAP signaling figures and at 40× magnification for all CellProfiler quantification figures.

Cell viability assays
Cells were seeded in 96-well plates at 3.5×10^3 cells /mL (200 µL per well) in DMEM, supplemented with 5% and 10% FBS, respectively, and 1% penicillin-streptomycin (whole media). The next day, cells were treated with BVdU diluted in DMSO from 1000 µM to 0.15 µM using a 3-fold serial dilution. One well was treated with DMSO as a control. After 5 days, cell viability was assessed using the CellTiter-Glo assay (Promega) per the manufacturer's protocol. Luminescence was measured using a plate reader (FLUOstar Omega). For the experiment in Figure S1D, cells were seeded in a 12-well plate at 5×10^4 cells per well in 2 mL of whole media. Every 48 hours, cells were harvested, viable cells were counted using a Vi-Cell XR cell counter and re-seeded at the original seeding density.

FACS competition assay
MEL-202$^{GNAQ(Q209L)}$ cells were stably transduced with lentivirus encoding *GNAQ*^{WT}, *GNAQ*^{Q209P}, or *GNAQ*$^{Q209P, L43A/L44A}$. Cells were selected with hygromycin (200 µg/mL, InVivoGen) for 7 days. MEL-202$^{GNAQ(Q209L)}$ cells and MEL-202$^{GNAQ(Q209L)}$ cells stably expressing the above-mentioned cDNA were transduced with lentivirus encoding (i) sg*NT* and *EGFP* or (ii) sg*GNAQ* #3 and mCherry. Forty-eight hours after transduction, *EGFP*-expressing cells were mixed in a 1:1 ratio with mCherry-expressing cells and analyzed by FACS on BD LSR-Fortessa. MEL-202$^{GNAQ(Q209L)}$ cells stably expressing Cas9 were stably transduced with lentivirus encoding (i) sg*NT* and *EGFP* or (ii) sg*GNAQ* #3 and mCherry, or (iii) sg*AP3S1* #3 and mCherry, followed by transduction with lentivirus encoding sg*AP3S2* #6 and BFP, 24 hours later. Forty-eight hours after transduction, *EGFP*-expressing cells were mixed in a 1:1 ratio with mCherry and/or *BFP*-expressing cells and analyzed by FACS on BD LSR-Fortessa. Data analysis was performed with FlowJo v10.8.1 software (Becton Dickinson & Company).

mRNA extraction and RT-qPCR
RNA was extracted and purified using the phenol-chloroform-isopropanol extraction method. RT-qPCR was performed using iTaq Universal SYBR Green One-Step kit (Bio-Rad), and amplification was measured using LightCycler 96 (Roche). Expression levels were calculated and normalized to *ACTB*.

CellProfiler analysis
Images were segmented using Cellpose3 plugin in CellProfiler4. Segmentation masks were generated using the Cellpose pre-trained model "cyto2" and object diameter of 180 (mask_cell) and 80 (mask_nuclei) pixels; minimum size of 100 (mask_cell) and 40 (mask_nuclei) pixels and flow threshold 0.8 (mask_cell) and 0.5 (mask_nuclei) to delineate the nucleus and cytoplasm. The
segmented masks and original images were then fed into CellProfiler to quantify the nuclear intensity of YAP and cytoplasmic GNAQ intensity.

YAP luciferase assay

293T cells were co-transfected with 8×GTIIC-luciferase (Addgene, #34615), TK-promoter driving *Renilla* luciferase (pRL-TK), and the indicated plasmids using TransIT-LT1 reagent. After 48h, the cells were processed for dual *Renilla* and firefly luciferase using the Dual-Luciferase Reporter Assay System (Promega, Cat #E1910).

TIDE analysis
Genomic DNA was isolated using the DNeasy Blood & Tissue Kit (QIAGEN, Cat# 69506). The region around the sgAP3S1 and sgAP3S2, AP3S1, and AP3S2 genomic DNA target sites was amplified by PCR (primers in Dataset S3). PCR amplicons were Sanger sequenced. The sequence trace files were uploaded to the TIDE web tool with the corresponding guide RNA sequence as input.

Animal Experiments
MEL-202$^{GNAQ(Q209L)}$ cells were stably transduced with lentivirus encoding *EGFP*, $GNAQ^{WT}$, $GNAQ^{Q209P}$, or $GNAQ^{Q209P,\ L43A/L44A}$. Cells were selected with hygromycin (200 μg/mL, InVivoGen) for 7 days. MEL-202$^{GNAQ(Q209L)}$ cells and MEL-202$^{GNAQ(Q209L)}$ cells stably expressing the above-mentioned cDNA were transduced with lentivirus expressing sg*ROSA26* or sg*GNAQ* #3 lentiCRISPR plasmids. The cells were selected with puromycin (1 μg/mL, InVivoGen) for 48 hours. 1×10^6 cells from each of the cell lines generated were injected intravenously into the tail vein of 8-week-old NSG mice (NOD.Cg-$Prkdc^{scid}$ $Il2rg^{tm1Wjl}$/SzJ, Strain #:005557; The Jackson Laboratory) by the Dana-Farber Cancer Institute Animal Resource Facility. The mice were euthanized after 5-10 weeks. The organs were harvested, fixed in 10% formaldehyde, and stored in 70% ethanol. All experiments were performed in compliance with federal laws and institutional guidelines and were approved by the Animal Care and Use Committee of the Dana-Farber Cancer Institute.

Immunohistochemistry
Livers harvested from mice were fixed overnight in 10% formalin and stored in ethanol 70%. Samples were submitted to the Brigham and Women's Hospital Pathology Core for paraffin embedding, sectioning, and hematoxylin and eosin and Sox10 staining and imaging. Immunohistochemistry was performed on the Leica Bond III automated staining platform using the Leica Biosystems Refine Red Detection Kit (Leica; DS9390). FFPE tissue sections were baked for 30 minutes at 60°C and deparaffinized (Leica AR9222) prior to staining. Primary antibodies were incubated for 30 minutes, visualized via DAB, and counterstained with hematoxylin (Leica DS9800). The slides were dehydrated for 30 minutes at 60°C and coverslipped using the HistoreCore Spectra CV mounting medium (Leica 3801733). Anti-SOX10 rabbit monoclonal primary antibody from was run at a 1:200 dilution with a 20M EDTA antigen retrieval (Leica ER2 AR9640). Whole slide imaging was performed on the Leica Aperio ScanScope AT Turbo. SOX10 positive cells were quantified using QuPath v.0.7.0 built-in "Positive cell detection" (Queen's University, Belfast, Northern Ireland)[3].

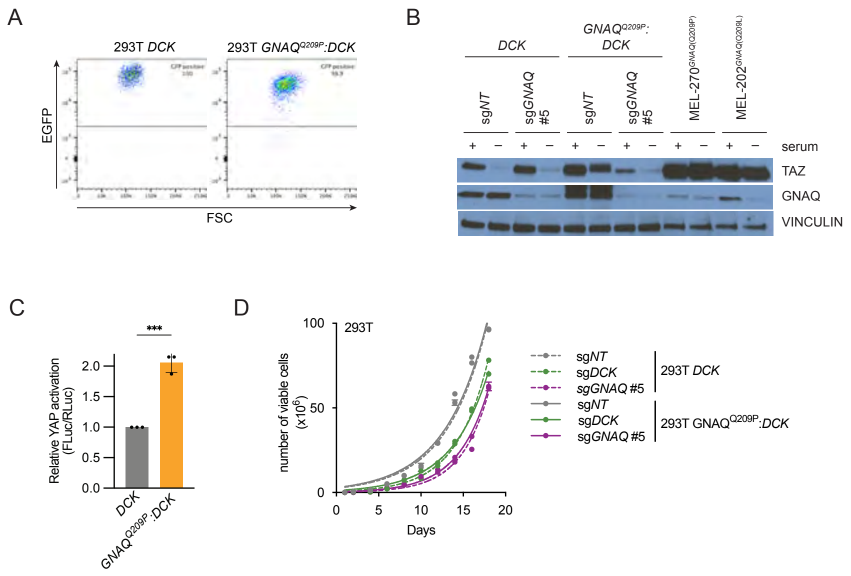

Fig. S1. Validation of 293T *DCK* and 293T *GNAQ*^{Q209P}*:DCK* cells for the positive selection screen.
(A) Fluorescence-activated cell sorting (FACS) analysis of 293T cells expressing *DCK* and *GNAQ*^{Q209P}*:DCK* constructs, which also encoded the green fluorescent protein (EGFP) (See Fig.1A) after FACS sorting. **(B)** Immunoblot analysis of 293T *DCK*, 293T *GNAQ*^{Q209P}*:DCK* cells transduced with indicated sgRNAs. +/- indicated cells cultured in the presence or absence of serum. **(C)** Activation of YAP-dependent promoter activation in indicated cell lines. **(D)** Growth of 293T *DCK* and 293T *GNAQ*^{Q209P}*:DCK* cells transduced with indicated sgRNAs. *n* = 1 biological replicate. For (C), an unpaired t-test was used to determine statistical significance. ns,

nonsignificant; *, P < 0.05; ***, P < 0.001; ****, P < 0.0001. Error bars represent the mean ± SEM of three technical replicates from one representative experiment.

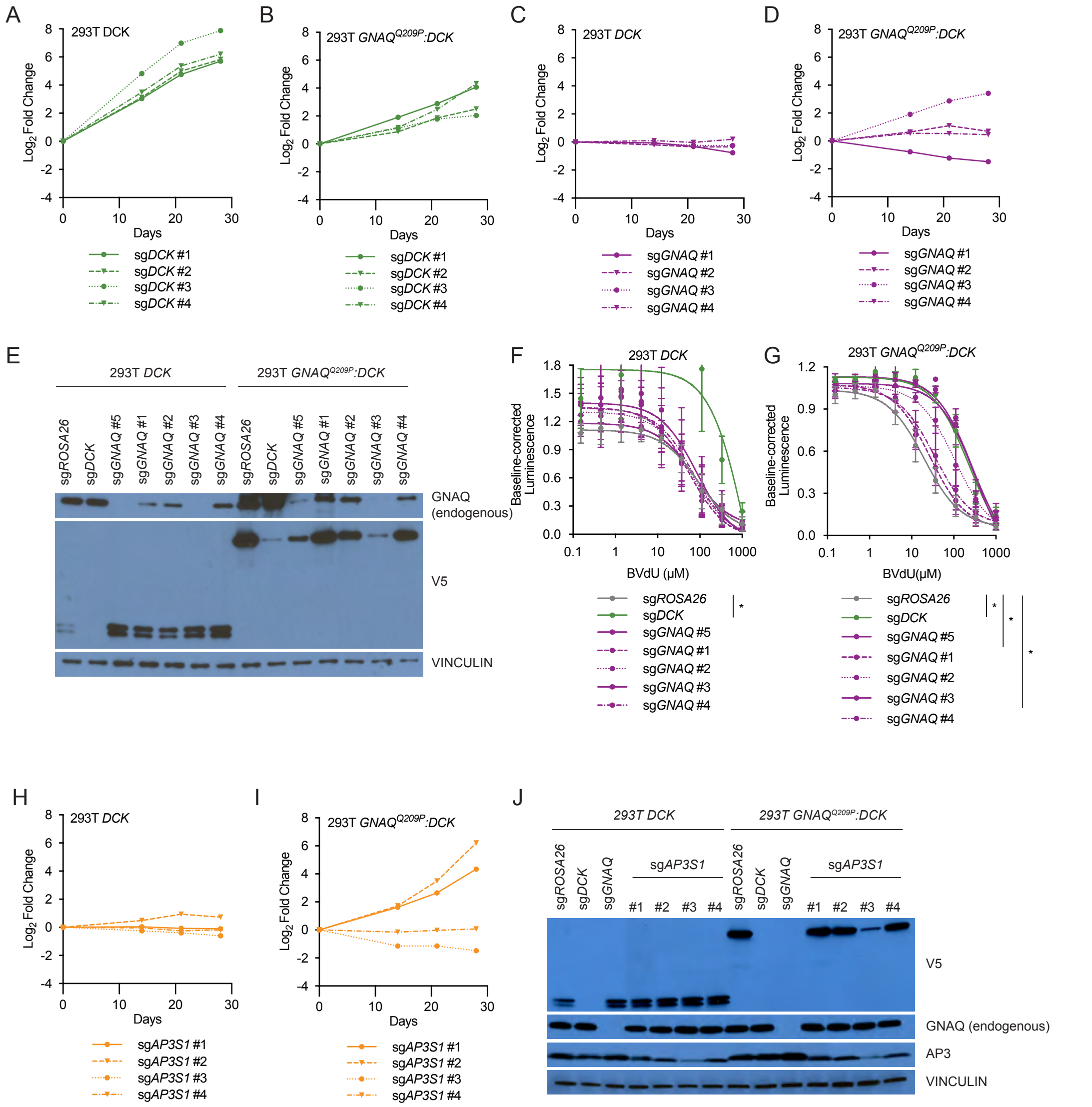

Fig. S2. Validation of sgRNAs from the whole genome Brunello sgRNA library targeting *DCK, GNAQ,* and *AP3S1*.
(A-D) Log_2 fold change (y-axis) of the indicated sgRNAs targeting *DCK* (A and B) and *GNAQ* at 14, 21, and 28 days (x-axis) of BVdU treatment relative to T0. **(E)** Immunoblot analysis of 293T *DCK*, 293T *GNAQ*^{Q209P}*:DCK* cells transduced with indicated sgRNAs. **(F, G)** Baseline-corrected luminescence of cells in (E) after 5 days of treatment with BVdU at the indicated concentrations. *n* = 3 biological replicates. **(H, I)** Log_2 fold change (y-axis) of indicated sgRNAs targeting *AP3S1* at 14, 21, and 28 days (x-axis) of BVdU treatment relative to T0. **(J)** Immunoblot analysis of 293T *DCK*, 293T *GNAQ*^{Q209P}*:DCK* cells transduced with indicated sgRNAs. Ordinary one-way ANOVA with multiple comparisons of AUC was used to determine statistical significance. ns, nonsignificant; *P < 0.05; ***P < 0.001; ****P < 0.0001. Error bars represent mean ± SEM.

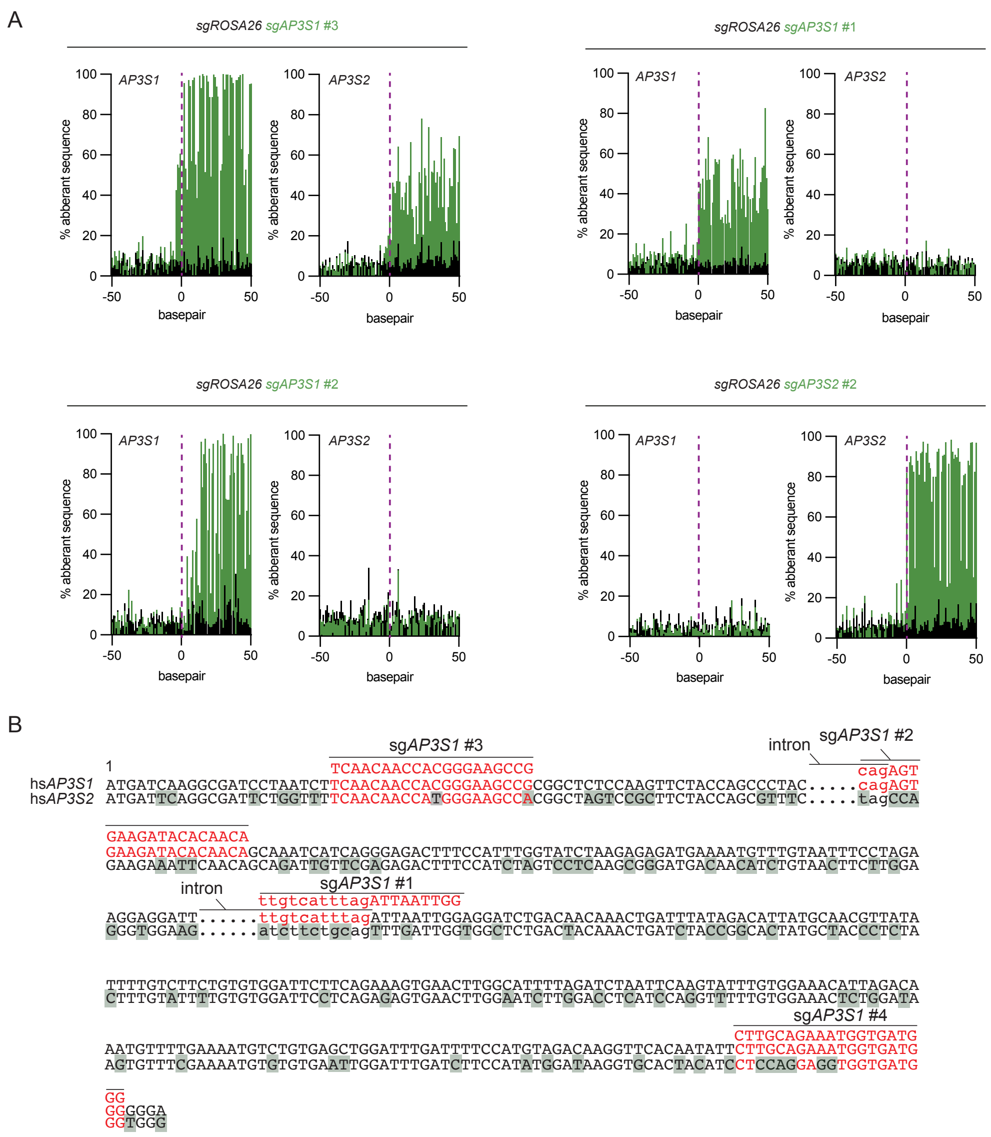

Fig. S3. sgAP3S1 #3 targets both *AP3S1* and *AP3S2.*
(A) TIDE analysis showing editing efficiency of sgRNAs targeting *AP3S1* or *AP3S2* in 293T *GNAQ*^{Q209P}*:DCK* measured by the percentage of aberrant nucleotides that are plotted along the sequence trace of the control (sg*ROSA26*) in black and the indicated sgRNAs targeting *AP3S1* or *AP3S2* in green. 0 = predicted editing site. **(B)** Alignment of *AP3S1* and *AP3S2* genomic DNA sequences to demonstrate overlap in the sequence of *AP3S1* targeting sgRNAs from the Brunello sgRNA library with *AP3S2.*

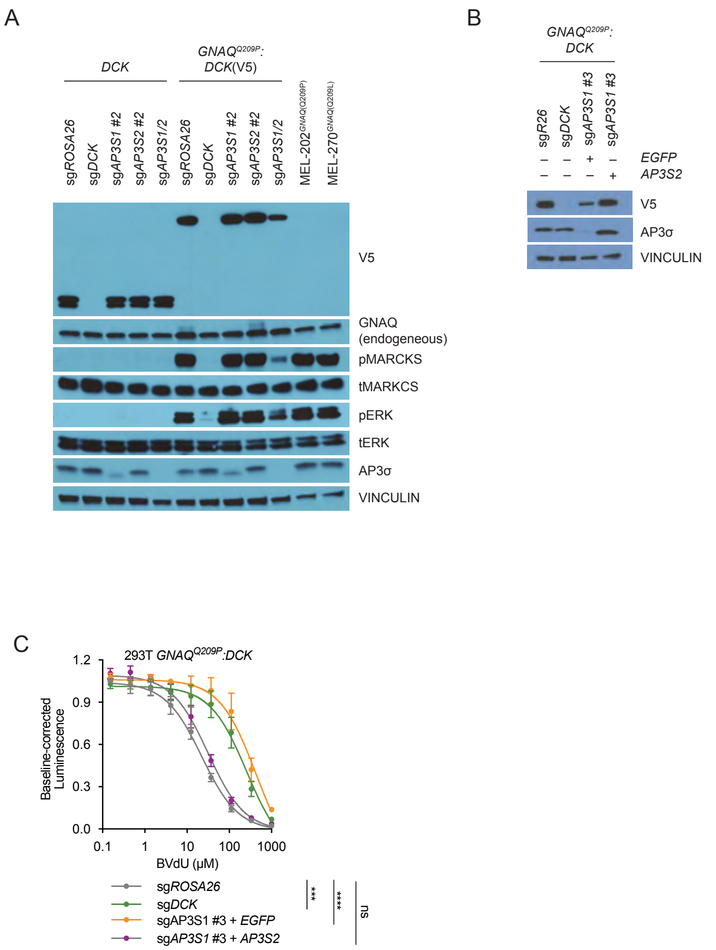

Fig. S4. *AP3S1* and *AP3S2* are necessary for Gαq* expression and downstream signaling. **(A)** Immunoblot analysis of 293T *DCK*, 293T *GNAQ*^{Q209P}*:DCK* cells transduced with indicated sgRNAs **(B)** Immunoblot analysis of 293T *GNAQ*^{Q209P}*:DCK* cells transduced with indicated sgRNAs and/or re-expression of sgRNA-resistant *AP3S2.* **(C)** Baseline-corrected luminescence of cells in (B) after 5 days of treatment with BVdU at the indicated concentrations. *n* = 3 biological replicates. Ordinary one-way ANOVA with multiple comparisons of AUC was used to determine statistical significance. ns, nonsignificant; *P < 0.05; ***P < 0.001; ****P < 0.0001. Error bars represent mean ± SEM.

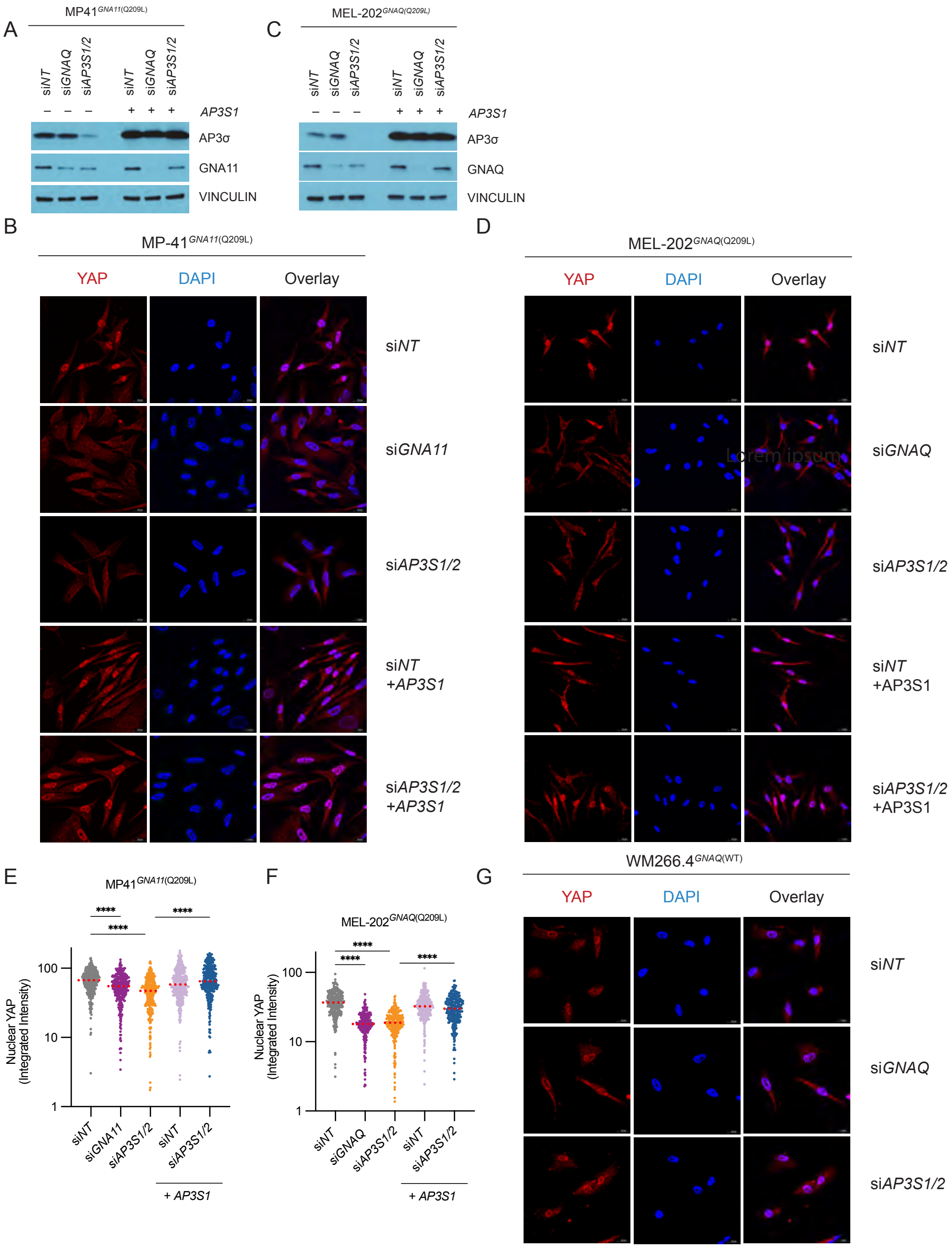

Fig. S5. AP-3σ is necessary for constitutively active Gαq/11* but not wild-type Gαq^{WT} mediated YAP signaling.

(A) Immunoblot analysis of MP41$^{GNA11(Q209L)}$ cells transfected with indicated siRNAs and/or re-expression of siRNA-resistant *AP3S1*. **(B)** Immunofluorescence staining for endogenous YAP (red) in MP41$^{GNA11(Q209L)}$ cells from (A). Scale bar, 25μm. (**C**) Immunoblot analysis of MEL-202$^{GNAQ(Q209L)}$ cells transfected with indicated siRNAs and/or re-expression of siRNA-resistant *AP3S1*. **(D)** Immunofluorescence staining for endogenous YAP (red) in MEL-202$^{GNAQ(Q209L)}$ cells from (C) transfected with the indicated siRNAs. Scale bar, 25μm. **(E)** Intensity of nuclear YAP staining in MP41$^{GNA11(Q209L)}$ cells from (B). *n*=320 cells**. (F)** Intensity of nuclear YAP staining in MEL-202$^{GNAQ(Q209L)}$ cells from (D). *n*=264 cells. **(G)** Immunofluorescence staining for endogenous YAP (red) in WM266.4$^{GNAQ(WT)}$ cells from (Fig. 2A) transfected with indicated siRNAs. Scale bar,

25μm. Ordinary one-way ANOVA with multiple comparisons was used to determine statistical significance. ns, nonsignificant; *P < 0.05; ***P < 0.001; ****P < 0.0001. Error bars represent mean ± SEM. si*NT:* control siRNA**.**

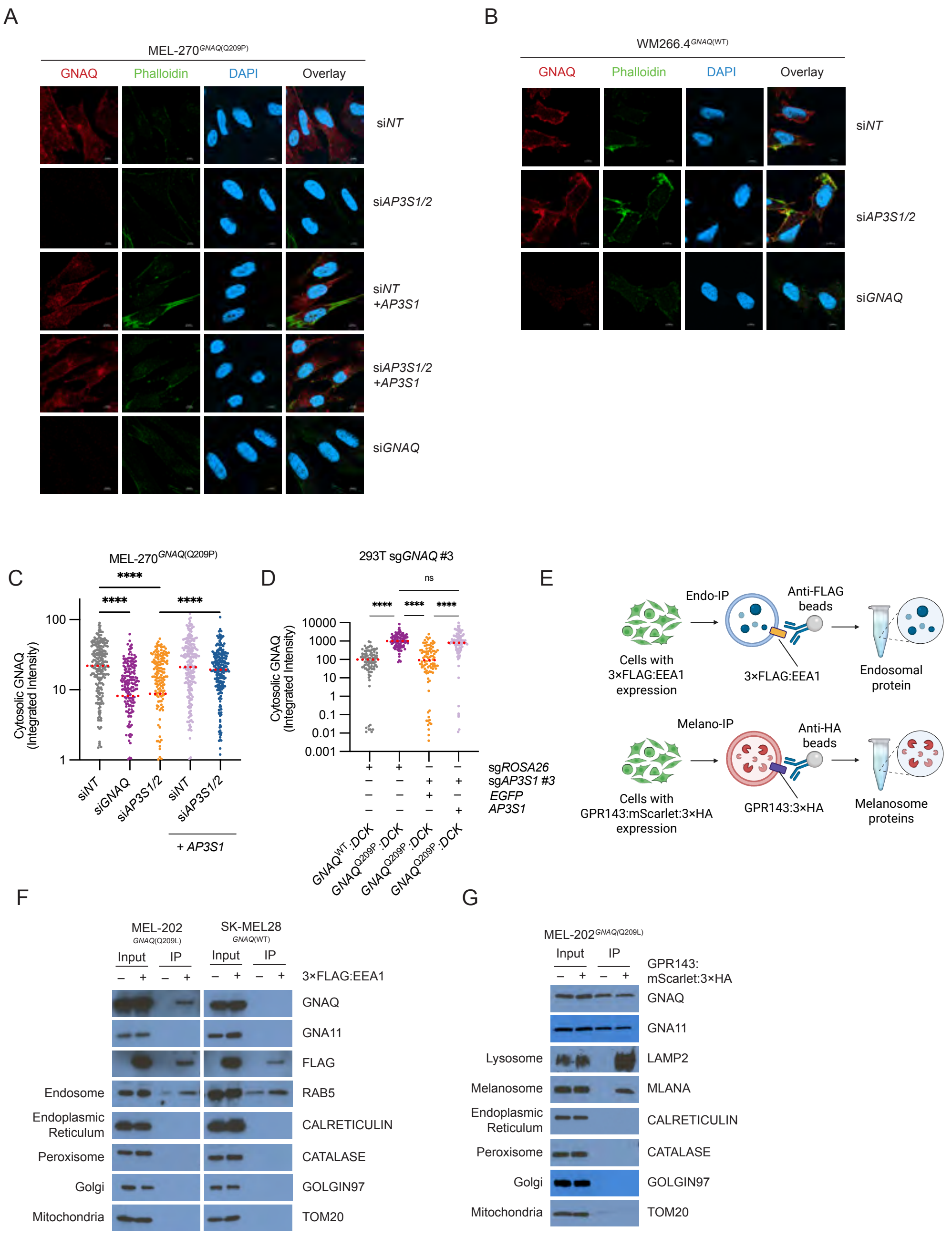

Fig. S6. Constitutively active, but not wild-type Gαq localizes to endosomes but not to melanosomes.

(A) Immunofluorescence staining for endogenous Gαq* (red) in MEL-270$^{GNAQ(Q209P)}$ cells transfected with the indicated siRNAs and/or re-expression of siRNA-resistant *AP3S1*. Scale bar, 5 μm. **(B)** Immunofluorescence staining for endogenous Gαq^{WT} (red) in WM266.4$^{GNAQ(WT)}$ cells transfected with the indicated siRNAs. Scale bar, 5 μm. **(C)** Intensity of cytoplasmic Gαq* staining in MEL-270$^{GNAQ(Q209P)}$ cells from (A). *n*=232 cells. **(D)** Intensity of cytoplasmic Gαq staining in cells from (Fig. 3D) *n*=92 cells. **(E)** Schematic representation of Endo-IP and Melano-IP method workflow. **(F)** Immunoblot analysis of endosomes purified (IP) by the Endo-IP method (E) from MEL-202$^{GNAQ(Q209L)}$ and SK-MEL28$^{GNAQ(WT)}$ cells lacking (-) or expressing exogenous 3xFLAG:EEA1 (+). **(G)** Immunoblot analysis of melanosomes purified (IP) by the Melano-IP method (E) from MEL-202$^{GNAQ(Q209L)}$ cells lacking (-) or expressing exogenous

GPR143:mScarlet:3XHA (+). Repeated measures of one-way ANOVA with multiple comparisons were used to determine statistical significance. ns, nonsignificant; *P < 0.05; ***P < 0.001; ****P < 0.0001. Error bars represent mean ± SEM. si*NT:* control siRNA**.**

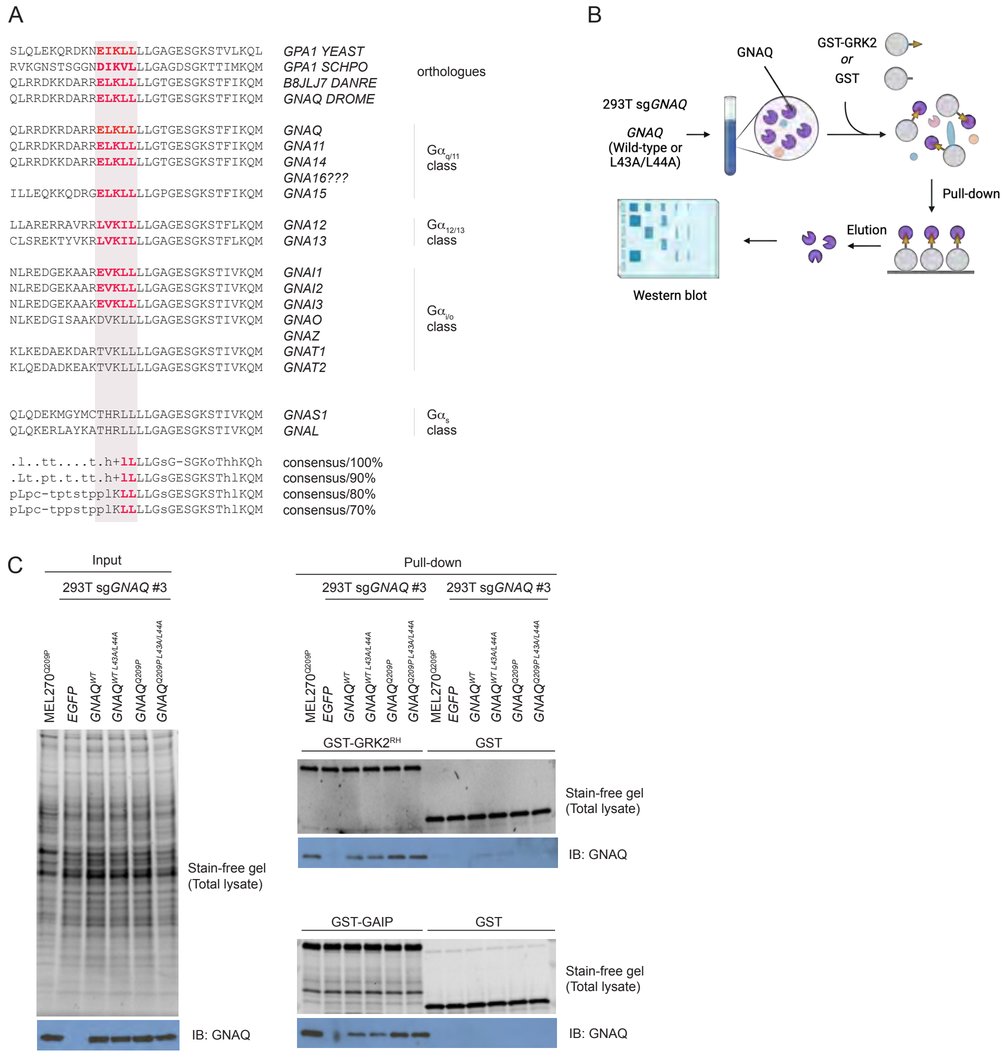

Fig. S7. L43 and L44 in the dileucine motif are not necessary for Gαq to bind to downstream effector proteins.
(A) Protein sequence showing dileucine sorting signal (red) conserved in different classes of Gα proteins as well as Saccharomyces cerevisiae (YEAST), Schizosaccharomyces pombe (SCHPO), Danio rerio (DNARE)/Zebrafish and Drosophila melanogaster (DROME) $G\alpha_{q/11}$ orthologs **(B)** Schematic representation of the workflow of GST-pull down assay. **(C)** Immunoblot assay showing GST-pull down of Gαq with GST-GAIP and GST-GRK2^{RH} using whole cell lysates from indicated cells. All indicated exogenous *GNAQ* cDNA and mutants are resistant to sg*GNAQ* #3.

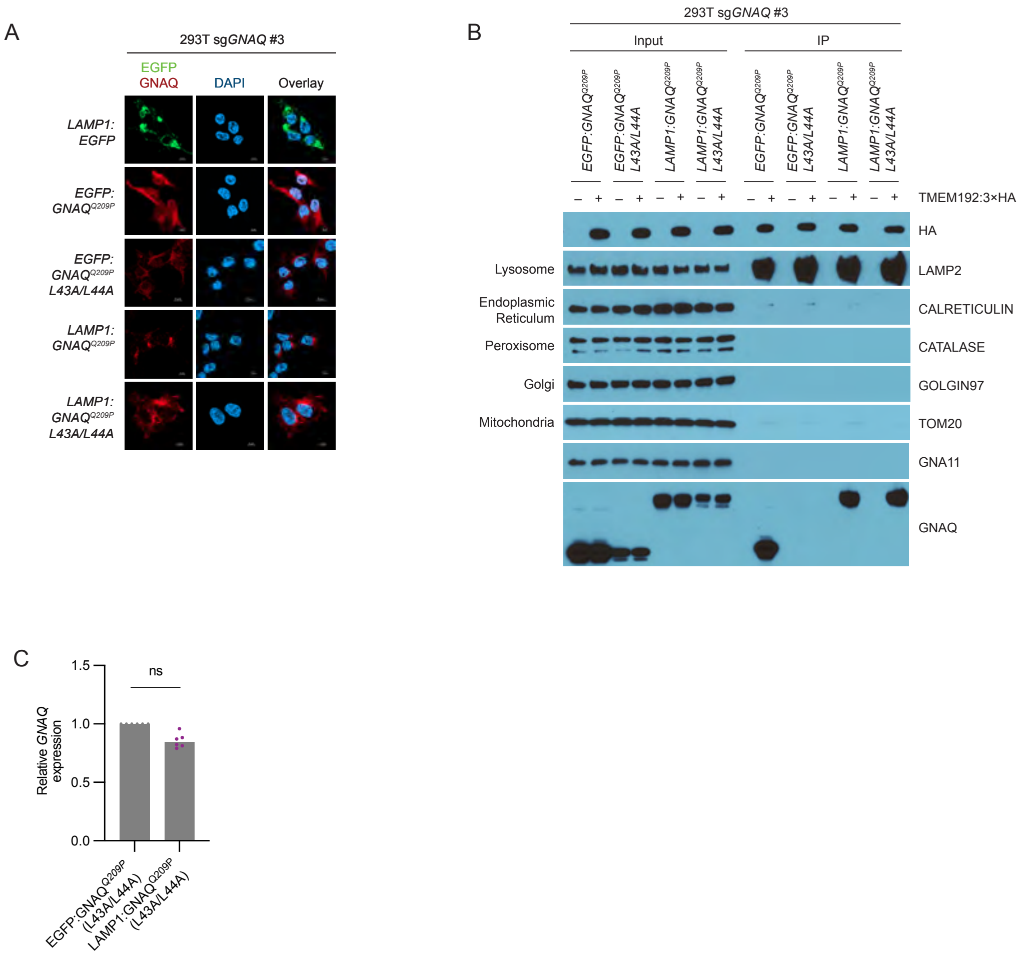

Fig. S8. LAMP1:Gαq* and LAMP1:Gαq$^{*, L43AL44A}$ localize to the lysosome.
(A) Immunofluorescence staining for Gαq in 293T sg*GNAQ* #3 cells expressing indicated cDNA. Scale bar, 5 μm. **(B)** Immunoblot analysis of lysosomes purified (IP) by the Lyso-IP method (Fig. 3E) from indicated cells lacking (-) or expressing exogenous TMEM192:3xHA (+). **(C)** Relative mRNA expression of indicated cDNA in 293T sg*GNAQ* #3 cells expressing indicated cDNA. All indicated exogenous *GNAQ* mutants and fusions are resistant to sg*GNAQ* #3.

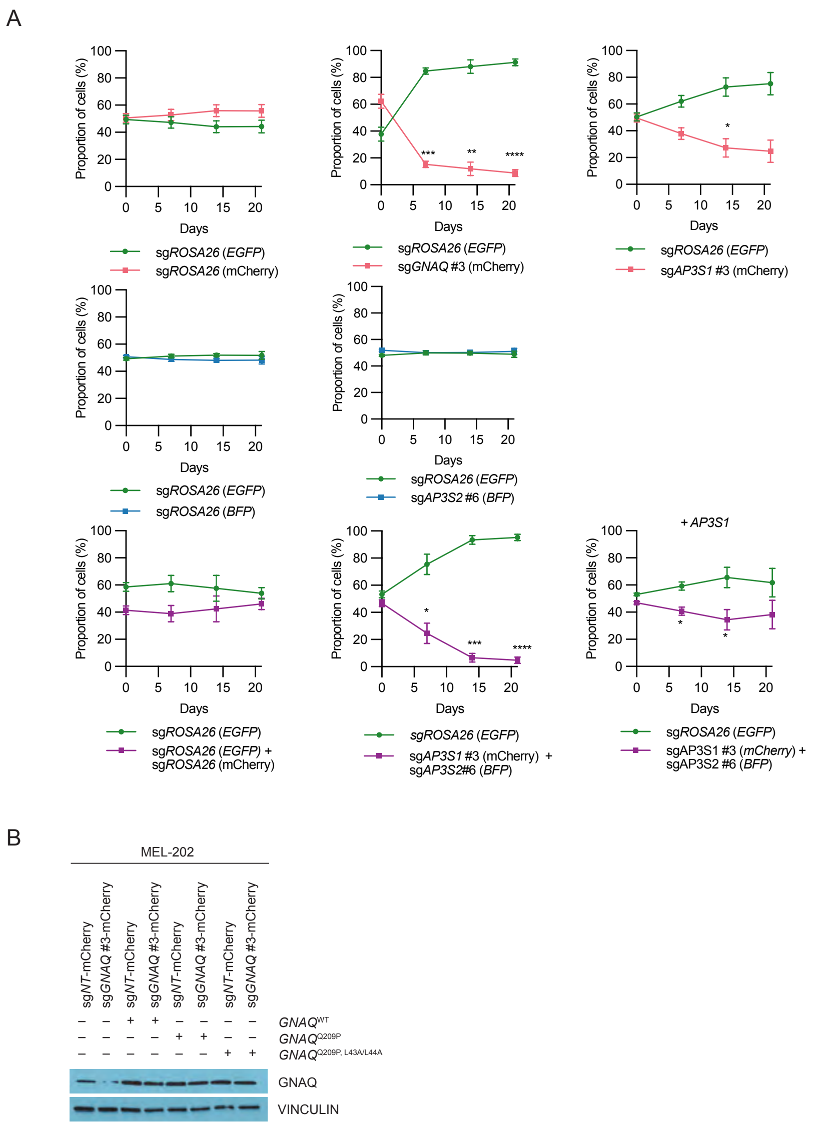

Fig. S9. AP-3σ is necessary for the growth of MEL-202 uveal melanoma cell line.
(A) The percentage of each cell type as determined by the FACS competition assay at the indicated days. Two-way ANOVA with multiple comparison test were used to determine statistical significance. ns, nonsignificant; *P < 0.05; ***P < 0.001; ****P < 0.0001. Error bars represent mean ± SEM. **(B)** Immunoblot of MEL-202$^{GNAQ(Q209L)}$ cells expressing indicated cDNAs.

Dataset S1. Results from positive selection whole genome CRISPR screen performed in 293T *DCK* and 293T $GNAQ^{Q209P}$*:DCK* cells.

Dataset S2. Gene Ontology (GO) cellular component analysis of top 100 genes enriched at day 28 in the 293T $GNAQ^{Q209P}$*:DCK* arm of the screen.

Dataset S3. Oligonucleotides

Dataset S4. Resource table

SI References

1. Brinkman, E.K., Chen, T., Amendola, M. & van Steensel, B. Easy quantitative assessment of genome editing by sequence trace decomposition. *Nucleic Acids Res* **42**, e168 (2014).
2. Maziarz, M. *et al.* Atypical activation of the G protein Galpha(q) by the oncogenic mutation Q209P. *J Biol Chem* **293**, 19586–19599 (2018).
3. Bankhead, P. *et al.* QuPath: Open source software for digital pathology image analysis. *Sci Rep* **7**, 16878 (2017).
